# Embryo morphology and live birth in the United States

**DOI:** 10.1016/j.xfre.2022.02.006

**Published:** 2022-02-23

**Authors:** Michael S. Awadalla, Jacqueline R. Ho, Lynda K. McGinnis, Ali Ahmady, Victoria K. Cortessis, Richard J. Paulson

**Affiliations:** a Division of Reproductive Endocrinology and Infertility, Department of Obstetrics and Gynecology, Keck School of Medicine, University of Southern California, Los Angeles, California; b Department of Preventive Medicine, Keck School of Medicine, University of Southern California, Los Angeles, California

**Keywords:** In vitro fertilization, embryo morphology, cleavage stage, blastocyst stage, live birth

## Abstract

**Objective:**

To determine the best-fit live birth rate per embryo based on maternal age, embryo stage, and embryo morphology.

**Design:**

Retrospective data analysis.

**Setting:**

Fertility clinics.

**Patient(s):**

The patients included were treated with in vitro fertilization in the United States at clinics reporting data to the Society for Assisted Reproductive Technology Clinic Outcomes Reporting System. We analyzed live birth data of unbiopsied autologous cleavage and blastocyst stage embryos for cycles started from 2016 through 2018. The analysis included 223,377 embryo transfers with a total of 336,888 embryos.

**Intervention(s):**

None.

**Main Outcome Measure(s):**

Live birth rate per embryo and rate of multiple gestations per pregnancy.

**Result(s):**

At the mean maternal age of 34 years, fresh embryos produced live birth rates of 19%, 38%, 26%, and 27% for embryos aged 3, 5, 6, and 7 days, respectively. At the age 34 years, live birth rates for day 5 fresh embryos by overall morphology grade were 43% for good, 30% for fair, and 21% for poor. For the transfer of 2 fresh day 5 blastocysts, the rate of multiple gestations per pregnancy was 47% at 25 years old, 44% at 30 years old, 35% at 35 years old, and 23% at 40 years old.

**Conclusion(s):**

The analysis of pregnancy data in the Society for Assisted Reproductive Technology database can be used to calculate live birth rates per embryo based on maternal age, embryo age, and morphology. This information can be used for evidence-based decision making, quality control, and planning multicenter studies.

**Discuss:** You can discuss this article with its authors and other readers at https://www.fertstertdialog.com/posts/xfre-d-22-00002

Understanding how maternal age, embryo morphology, and embryo developmental stage affect the live birth rate (LBR) per embryo is important for understanding normal in vitro embryo development. Knowing the LBR per embryo is a fundamental starting point to determine the risk of twins and higher-order multiples for a planned embryo transfer. Although the American Society for Reproductive Medicine has published guidance on the maximum number of embryos to transfer, there is limited quantitative data on the risk of multiples for a planned embryo transfer (1). Many published studies are limited by small sample sizes and statistical methods that report odd ratios rather than actual rates of LBR per embryo. The LBR per embryo can be used as an intermediate endpoint to evaluate in vitro fertilization stimulation protocols and to track embryo culture quality. The LBR per embryo can be used in data analysis to control for maternal age, embryo stage, morphology, number of embryos transferred, and clinic. This will allow the design of better multicenter studies on frozen embryo transfer protocols.

Logistic regression has commonly been used to determine best-fit LBRs for embryos (2, 3, 4, 5, 6, 7, 8). This technique facilitates high-order multivariate analysis of smaller datasets that cannot sustain stratified analysis, but it requires model assumptions that may not be warranted in an analysis addressing the transfer of multiple embryos with different morphologies (3). An alternative approach that we previously described is the use of linear algebra to model embryo transfers (9, 10). Although more commonly used for engineering applications, linear algebra is suitable for modeling embryo transfers and avoids many of the challenges encountered with logistic regression.

The primary objective of this study was to determine the best-fit LBR for specific unbiopsied human embryos created through in vitro fertilization based on embryo stage and morphology. The secondary objective was to fit a model for predicting rates of live birth and multiple gestations after a multiple-embryo transfer.

## Materials and methods

### Study Population and Data

**Figure 1:**
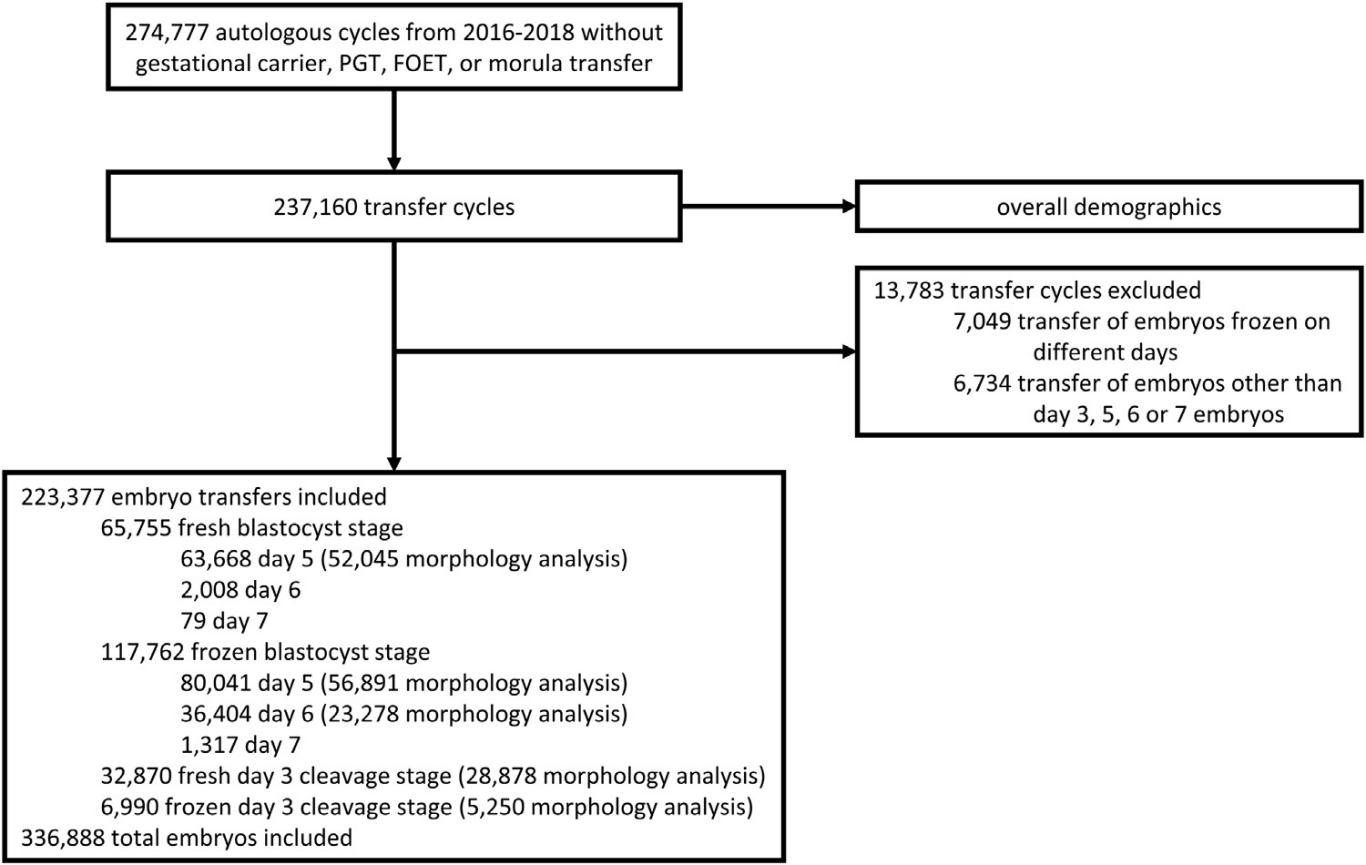
Flow Diagram of Included and Excluded Cycles. PGT = preimplantation genetic testing, FOET = frozen-oocyte embryo transfer.

Data were provided retrospectively by the US Society for Assisted Reproductive Technology (SART) from the Clinic Outcomes Reporting System for autologous fresh and frozen embryo transfer cycles started from 2016 through 2018. Cycles were excluded if they used a gestational carrier, preimplantation genetic testing, frozen-oocyte embryo transfer, or transfer of a morula-stage embryo. This subset of data consisted of 237,160 embryo transfer cycles. For the analysis of morphology and LBRs, we further excluded transfers of embryos frozen on different days to simplify the analysis. We excluded transfers of embryos other than day 3, 5, 6, or 7 embryos due to limited data on these tranfers. The remaining 223,377 embryo transfers, with a total of 336,888 embryos, were included in the final analysis (Fig. 1). This study was approved by the University of Southern California institutional review board (HS-19-00261) and the SART research committee.

Detailed morphological analysis was performed for the transfers with embryo morphology data available. Consensus guidelines for the assessment of embryo morphology have been presented by the SART in 2010 (11) and the Istanbul consensus workshop in 2011 (12). The standardized grading for cleavage-stage embryos used by the SART includes cell number (1, 2, 3, 4, 5, 6, 7, 8, or >8), fragmentation (0%, 1%–10%, 11%–25%, or >25%), and symmetry (perfect, moderately asymmetric, or severely asymmetric). The standardized grading for blastocysts used by the SART is similar to that proposed by Gardner and Schoolcraft (13), but does not explicitly use that system (11). The SART allows clinics to give an overall blastocyst grade of good, fair, or poor, but does not specify further on how clinics must assign these grades. The expansion stage is specified as early, expanded, or hatching blastocyst. The inner cell mass (ICM) and trophectoderm (TE) are each given a score of good, fair, or poor.

### Linear Algebra Model and Moving Age Groups

We have previously described the use of linear algebra and moving age groups to determine the best-fit LBRs per embryo based on stage and morphology (9, 10). Briefly, each embryo transfer is modeled as an equation, and the best-fit LBRs are determined by solving all the equations concurrently. For example, for a transfer of 3 embryos resulting in 1 live birth, the best-fit LBR would be 33.3% per embryo. We repeated the analysis at each integer value of maternal age by considering an age group of size varying from 1 to 9-years centered on that age. A more detailed discussion of these methods is included in the Supplemental Methods (available online).

### Statistical Methods

We used stratification as the primary means of controlling for potentially confounding variables. Data were stratified by transfer type (fresh or frozen) and day of the embryo (day 3, 5, 6, or 7). We used moving age groups to smooth the data and stratify by maternal age. We had previously determined that using moving age groups had a very small effect on the best-fit LBRs per embryo because the LBRs are approximately linear over small age ranges (9). Stratification by maternal age is important because age confounds the relationship between morphology and live birth. An analysis that controlled for age revealed that morphology had a lesser impact on the LBRs than seems apparent when age is not controlled (9). We used SAS (version 9.4; SAS Institute, Cary, NC) and Excel (version 2105; Microsoft Corp., Redmond, WA) to sort and filter the data. We used a custom program in MATLAB (version 9.5; MathWorks, Natick, MA) to analyze the data in moving age groups and to perform the linear algebra (14).

We used linear algebra to control for the number of embryos transferred and the quality of each embryo. Specifically, we assumed that each embryo implants independently of the other embryos that are transferred concurrently (15). Blastocyst (day 5, 6, and 7 embryos) quality is expressed by an overall quality grade (good, fair, or poor), ICM quality (good, fair, or poor), TE quality (good, fair, or poor), and expansion stage (early, expanded, or hatching blastocyst). Day 3 cleavage-stage embryo quality is expressed by cell number on day 3 (4, 5, 6, 7, 8, or >8 cells) and fragmentation percentage (0%, 1%–10%, or >10%).

### Live Birth Rate per Embryo by Maternal Age, Embryo Stage, and Fresh/Frozen Transfer

Our primary analysis of LBR per embryo was performed without considering embryo morphology. In this analysis, we considered LBR per embryo to be dependent on only maternal age at oocyte retrieval, embryo stage (defined as the day of culture on which the embryo was transferred or cryopreserved), and the type of embryo transfer cycle (fresh or frozen).

We performed several subgroup analyses. We evaluated the LBR per embryo for day 3 and day 5 embryos stratified by single vs. multiple-embryo transfer. We also performed detailed morphology analysis for embryos in the following categories: day 3 fresh, day 3 frozen, day 5 fresh, day 5 frozen, and day 6 frozen (Fig. 1). Details on the transfers included in these subgroup analyses are provided in the Supplemental Methods.

### Embryo Transfer Model for Predicting Risk of Multiples

We have previously described a model for predicting the risk of multiple gestations after transfer of multiple embryos (9, 10, 16). This model allows for inclusion of quantitative measures of uterine receptivity and embryo competence. The uterine receptivity is expressed as a percentage of transfers in which the uterus is receptive to embryo implantation which can be anywhere from 0%–100% and is expressed as a number from 0 to 1. We generalized the concept of uterine receptivity to include all factors that affect all embryos transferred concurrently and termed this as “universal factors fraction” (UNI). The UNI is determined as a best-fit value based on the rates of singleton and multiple gestations that result after transfer of multiple embryos. The basic principle for making this determination is the assumption that if uterine receptivity or other factors such as embryo transfer technique are not favorable, none of the transferred embryos will implant (Supplemental Fig. 1, available online).

For example, if 2 embryos each have an overall LBR per embryo of 25% and the UNI is only 50%, then each embryo has a 0% chance of live birth when the uterus is not receptive and a 50% chance of live birth of when the uterus is receptive. The chance of twins when both embryos are transferred is then 0.5 × 0.5^2^, which is 0.125 or 12.5% (Supplemental Fig. 1).

We used a custom computer program to find the best-fit UNI for the following groups of embryo transfers: fresh day 3, frozen day 3, fresh day 5, and frozen day 5 (17). We compared the predicted rates of multiples to the actual rates to evaluate the model fit in subgroups based on age for day 3 fresh, day 5 fresh, and day 5 frozen transfers.

## Results

### Demographics

The mean maternal age at oocyte retrieval was 33.9 years (SD, 4.5 years), and the mean maternal body mass index was 26.7 kg/m^2^ (SD, 6.7 kg/m^2^). Additional demographics and cycle characteristics are provided in Supplemental Table 1 (available online).

### Live Birth Rate per Embryo by Maternal Age, Embryo Stage, and Fresh/Frozen Transfer

**Figure 2:**
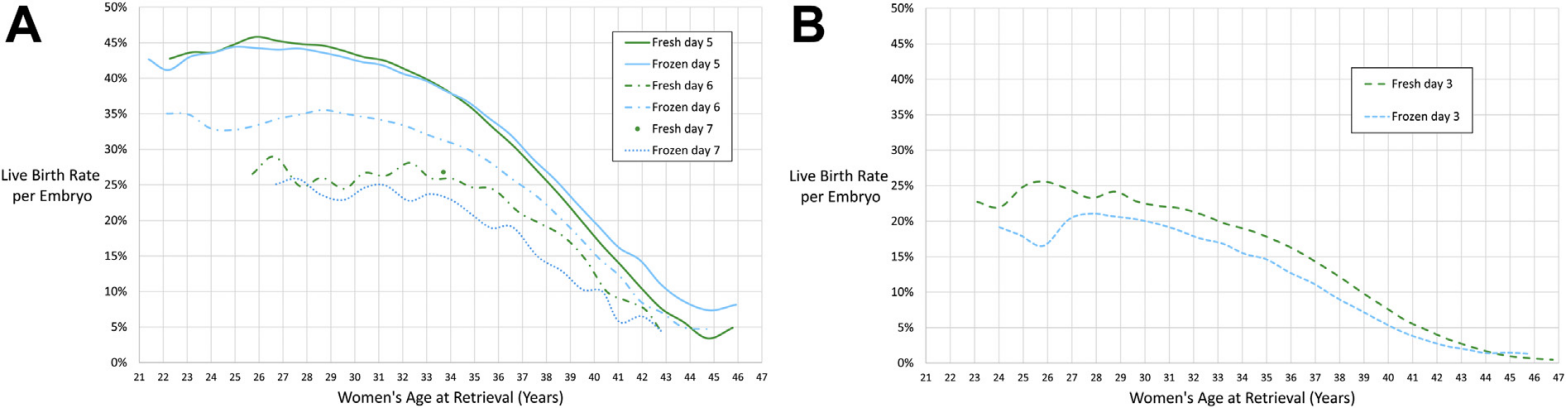
Best-fit live birth rate per embryo using 5-year moving age groups for (**A**) blastocyst and (**B**) cleavage-stage transfers. Age groups with fewer than 100 transfers are excluded.

The best-fit LBR per embryo was highest for day 5 blastocysts in younger patients. The LBR for day 5 embryos was similar for fresh and frozen embryos in younger patients and higher for frozen embryos in patients aged >35 years. The next highest LBR per embryo was in the frozen day 6, followed by fresh day 6 embryos. Frozen day 7 embryos had the lowest LBRs, and there was not enough data on fresh day 7 embryos to compare these to other groups (Fig. 2A and Supplemental Table 2 [available online]). For day 3 cleavage-stage embryos, fresh embryos had slightly higher LBRs than frozen embryos. At the mean maternal age of 34 years, the best-fit LBRs per embryo were 38% for fresh day 5, 38% for frozen day 5, 26% for fresh day 6, 31% for frozen day 6, 27% for fresh day 7, and 23% for frozen day 7 embryos. For cleavage-stage embryos at 34 years old, the LBRs were 19% for fresh day 3 embryos and 15% for frozen day 3 embryos (Fig. 2B).

### Single vs. Multiple-Embryo Transfer

The LBRs were higher for embryos transferred in single embryo transfers than those transferred in multiple-embryo transfers. The best-fit LBRs per fresh day 5 embryo at the age of 34 years were 45% for single embryo transfer and 35% for multiple-embryo transfers. Similar differences were seen in the LBRs of day 3 embryos and day 5 frozen embryos (Supplemental Figs. 2–4, available online).

#### Day 5 and 6 blastocyst detailed morphology analysis

**Figure 3:**
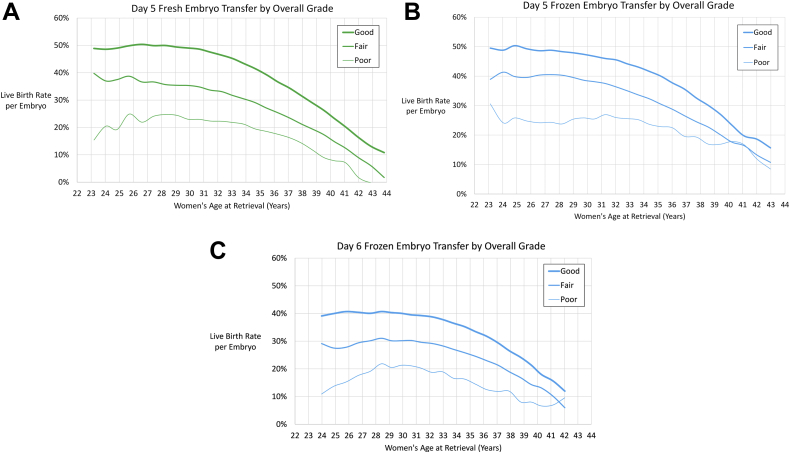
Best-fit live birth rate per blastocyst based on overall grade for (**A**) day 5 fresh transfers, (**B**) day 5 frozen transfers, and (**C**) day 6 frozen transfers. Seven-year moving age groups were used.

Embryo morphology distribution is shown in Supplemental Figures 5 and 6 (available online). The most common overall grade assigned to fresh day 5 blastocysts was good (76%), followed by fair (21%) and poor (3%). The ICM and TE were assigned the same quality score approximately 80% of the time. Similar trends were seen for day 5 frozen and day 6 embryos. At the mean age of 34 years, embryos with an overall grade of good had approximately 10% higher LBRs than fair quality embryos when controlling for age, transfer type, and embryo stage (Fig. 3A to C). The LBRs for day 5 and day 6 blastocysts by age, expansion stage, ICM quality, TE quality, and combinations of ICM and TE quality are given in Supplemental Figures 7–18 (available online) and Supplemental Tables 3–17 (available online).

#### Day 3 cleavage stage detailed morphology analysis

**Figure 4:**
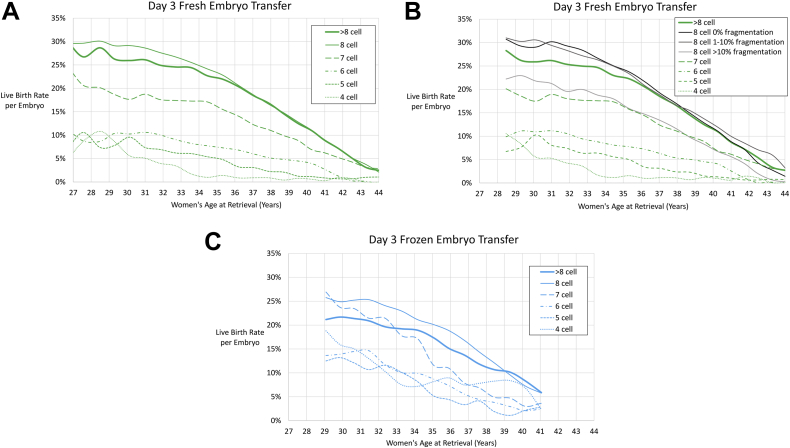
Best-fit live birth rates based on cleavage-stage embryo morphology for (**A**) day 3 fresh, (**B**) day 3 fresh with fragmentation, and (**C**) day 3 frozen embryo transfers. Seven-year moving age groups were used.

The most common day 3 embryo cell number was 8 cell (48%), followed by >8 cell (18%), 6 cell (12%), 7 cell (11%), 5 cell (6%), and 4 cell (6%). The highest fresh day 3 embryo LBRs at 34 years were for 8 cell embryos (24%), followed by >8 cell (23%), 7 cell (17%), 6 cell (8%), 5 cell (5%), and 4 cell (1%) embryos. The 8 cell embryos with lower fragmentation percentages had higher LBRs. Similar trends were seen for frozen day 3 embryos as shown in Figure 4A to C and Supplemental Tables 18–20 (available online).

### Embryo Transfer Model for Predicting Risk of Multiples

Considering only multiple-embryo transfers, the best-fit UNI using previously described methods (16) was 0.52 for fresh day 3 embryo transfers, 0.48 for frozen day 3 transfers, 0.60 for fresh day 5 transfers, and 0.66 for frozen day 5 transfers. A UNI of 0.66 represents favorable factors for embryo implantation 66% of the time. Based on this model and a UNI of 0.70, predicted rates of multiple gestations based on the number of embryos and average LBR per embryo are given in Supplemental Figure 19 (available online). For example, the transfer of an embryo with a 30% LBR and an embryo with a 22% LBR would have an average LBR of 26% per embryo. Supplemental Figure 19 predicts a 42% LBR, with 23% of those deliveries being twins. This model is meant to account for multiple gestations resulting from separate zygotes since this is the most common type of multiple gestations and the most clinically relevant when determining an appropriate number of embryos to transfer.

#### Risk of multiples for day 3 fresh cleavage-stage transfer

Out of 9,160 fresh day 3 single embryo transfers, there were 1,424 live deliveries including 25 twin deliveries for a LBR of 15.5% per transfer and a twin rate of 1.8% per delivery. The multiple gestation rate (number of deliveries of twins or higher divided by the total number of deliveries) for day 3 fresh double embryo transfer was 26% at 25 years, 26% at 30 years, 22% at 35 years, and 13% at 40 years. The predictions based on the model were similar and are given for double and triple embryo transfers in Supplemental Figure 20 (available online).

#### Risk of multiples for day 5 fresh blastocyst transfer

Out of 37,288 fresh day 5 single embryo transfers, there were 16,394 live deliveries including 259 twin deliveries for a LBR of 44.0% per transfer and a twin rate of 1.6% per delivery. The multiple gestation rate for double embryo transfer was 47% at 25 years, 44% at 30 years, 35% at 35 years, and 23% at 40 years. Model predictions are given for comparison in Supplemental Figure 21 (available online).

#### Risk of multiples for day 5 frozen blastocyst transfer

Out of 52,038 frozen day 5 single embryo transfers, there were 23,288 live deliveries including 314 twin deliveries for a LBR of 44.8% per transfer and a twin rate of 1.3% per delivery. The multiple gestation rate for double embryo transfer was 43% at 25 years, 40% at 30 years, 34% at 35 years, and 24% at 40 years. Model predictions are given for comparison in Supplemental Figure 22 (available online).

## Discussion

### Use of Results

The LBRs per embryo reported have 5 main uses. The first use is in general patient counseling both before starting in vitro fertilization (IVF) stimulation and after completion of a fresh IVF stimulation cycle. The second use is for determining a safe number of embryos to transfer at one time. A third use is to evaluate the performance of an IVF stimulation protocol. For example, the sum of all the individual LBRs of all the embryos created could be used as a performance metric. The fourth use is for evaluating embryo culture performance. The summed LBRs of all the embryos at the cleavage and blastocyst stages could be used to quantitatively evaluate embryo culture performance. Lastly, the expected LBR per embryo transferred can be used to control for maternal age and embryo quality when evaluating frozen embryo transfer protocol performance.

### Observed Trends

We observed several interesting findings in specific subgroups of embryo transfers. In the day 5 fresh and frozen transfers, the highest LBRs per embryo were around 26 years. This may represent noise in the data or there may be lower LBRs per embryo at younger extremes of age due to increased rates of aneuploidy found in younger patients (18). The LBR per day 5 blastocyst was higher in frozen transfers compared to fresh transfers in ages over 40 years (Fig. 2A and Fig. 3A and B). Since this may be a result of selection bias, a prospective randomized controlled trial would need to be performed to evaluate this further. For the day 7 fresh embryo transfer group there were only 79 transfers and the average age was 33.7 years. There were 123 fresh day 7 embryos transferred (an average of 1.6 embryos per transfer) and 33 live births resulted for a LBR of 27% per embryo. As observed in a previous study (19), there were very low LBRs for cleavage-stage embryo transfers in older patients. Out of 356 day 3 fresh autologous single embryo transfers in women aged 45–52 years there were 0 live births. The live birth rate per embryo was similar from age 21 years to 34 years. After 34 years old the live birth rate per embryo decreased. This suggests that logistic regression may not be appropriate for analysis of data that spans the entire range of reproductive ages since the logit assumption is not met (Supplemental Discussion and Supplemental Figure 23).

### Limitations

Differential reporting of overall embryo grade may limit comparisons of studies on overall blastocyst grade. In this study, approximately 75% of blastocysts were scored as good overall grade. Studies using more stringent grading are expected to have higher LBRs for good-quality blastocysts (9). Our analysis of different combinations of ICM and TE quality was limited by few embryos graded with different reported quality for these measures. Our preferred use of a UNI of 0.70 for predicting risk of multiples is based on rounding the best-fit result of the most common embryo transfer type (0.66 for frozen day 5 transfers). This value of 0.70 was used in previous studies (9, 10, 20). Using a value close to 0.50 would help to correct for some underestimation of multiples risk for fresh day 3 transfers (Supplemental Fig. 20).

## Conclusion

Cleavage-stage embryos with 8 cells have the highest LBRs. Good-quality day 5 blastocysts in young patients have a LBR of approximately 50%. Blastocysts with good overall grades have approximately 10% higher LBRs than those with fair overall grades. Fresh and frozen day 7 embryos have a LBR of approximately 25% for ages <35 years. The twin rate after single cleavage or blastocyst stage embryo transfer is approximately 1.5%. The risk of twins after transfer of 2 fresh day 3 embryos is approximately 25% in women <35 years old. The risk of twins after transfer of 2 fresh or frozen day 5 embryos is approximately 40% in women <35 years old. The LBRs per embryo presented can be used to counsel patients, for embryo culture quality control, to evaluate performance of IVF stimulation, and to control for embryo quality in evaluating the performance of embryo transfer protocols.

## Supplementary data

Supplemental Figures

Supplemental Methods and Discussion
