# Supplementary material for: Embryo morphology and live birth in the United States
Source: F S Rep. 2022 Feb 23;3(2):131–7. doi: 10.1016/j.xfre.2022.02.006 (PMC9250116; doi:10.1016/j.xfre.2022.02.006)

**Supplemental Figures**

Embryo Morphology and Live Birth in the United States

Michael S. Awadalla, M.D., Jacqueline R. Ho, M.D., Lynda K. McGinnis, Ph.D., Ali Ahmady, Ph.D., Victoria K. Cortessis, Ph.D., Richard J. Paulson, M.D.

## Supplemental Figure 1


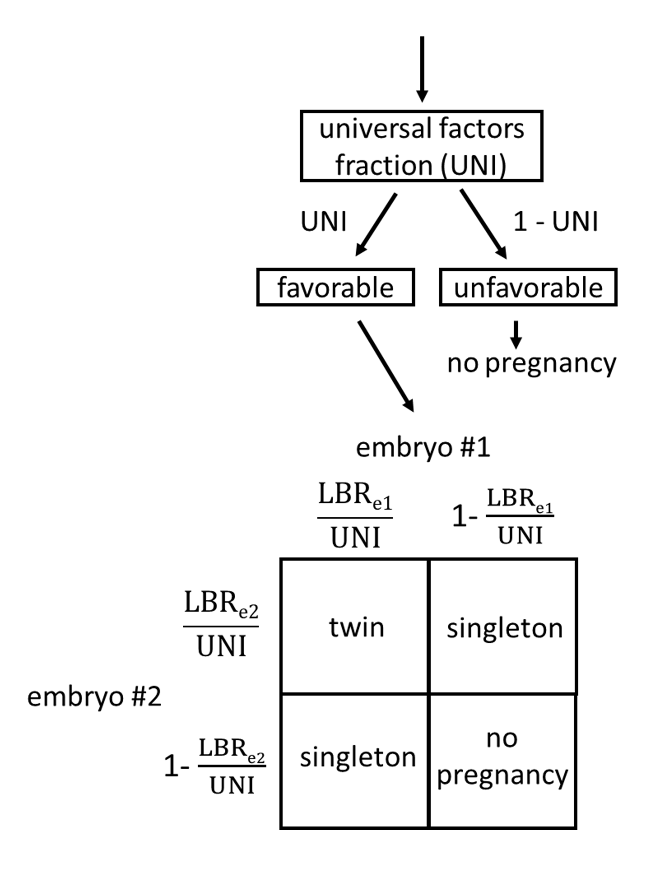


## Supplemental Figure 2


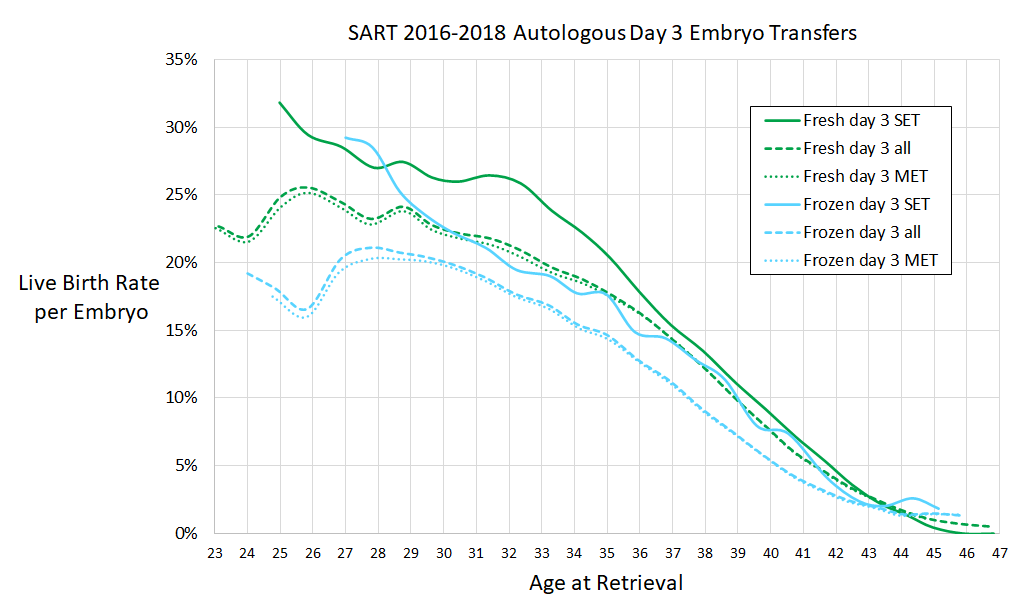


## Supplemental Figure 3


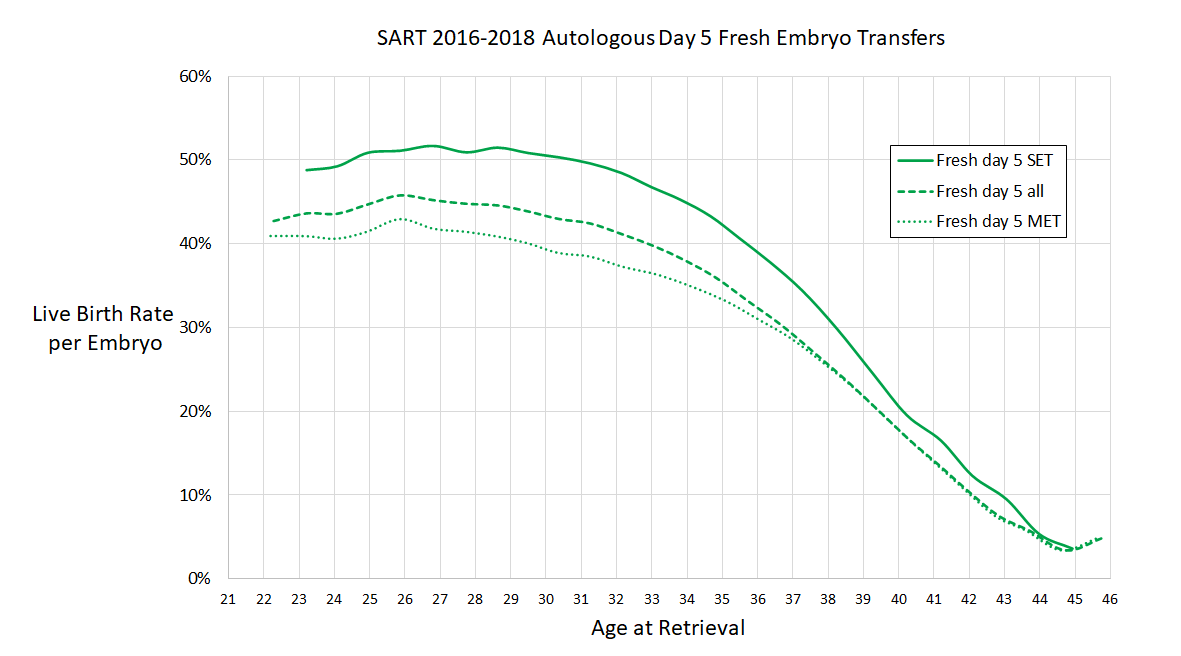


## Supplemental Figure 4


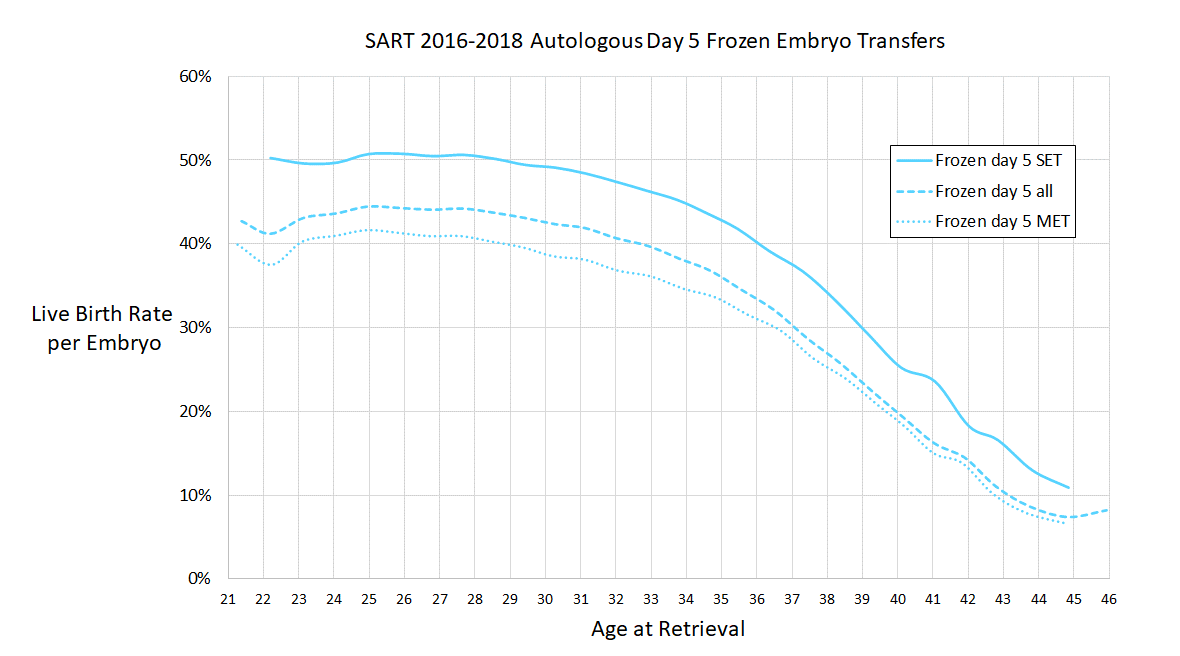


## Supplemental Figure 5


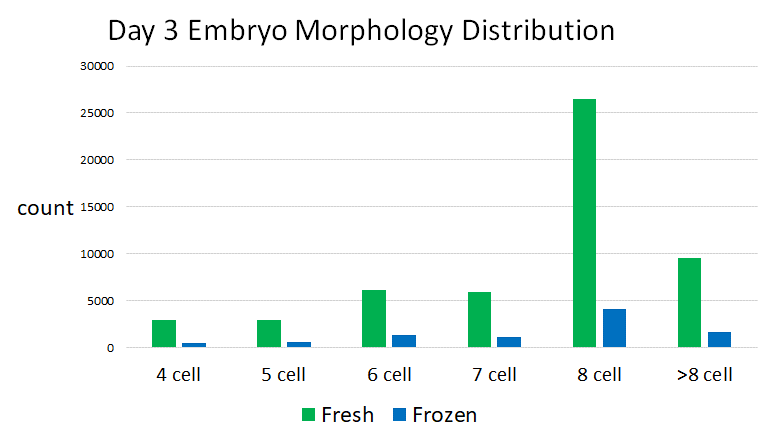


## Supplemental Figure 6


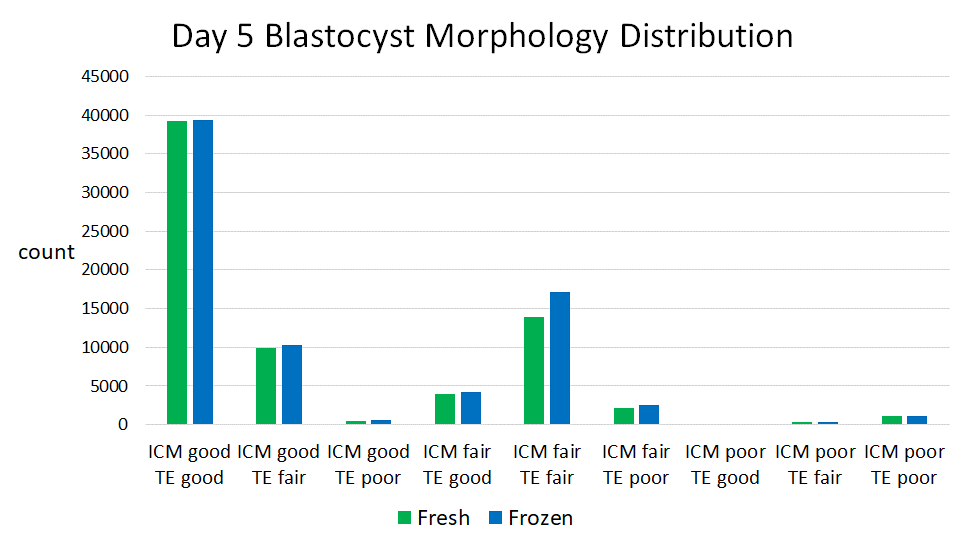


## Supplemental Figure 7


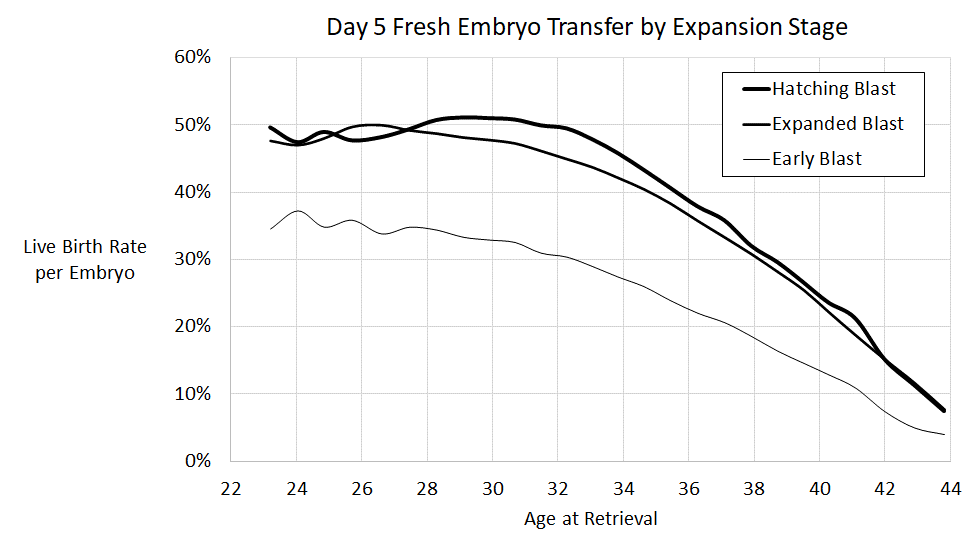


## Supplemental Figure 8


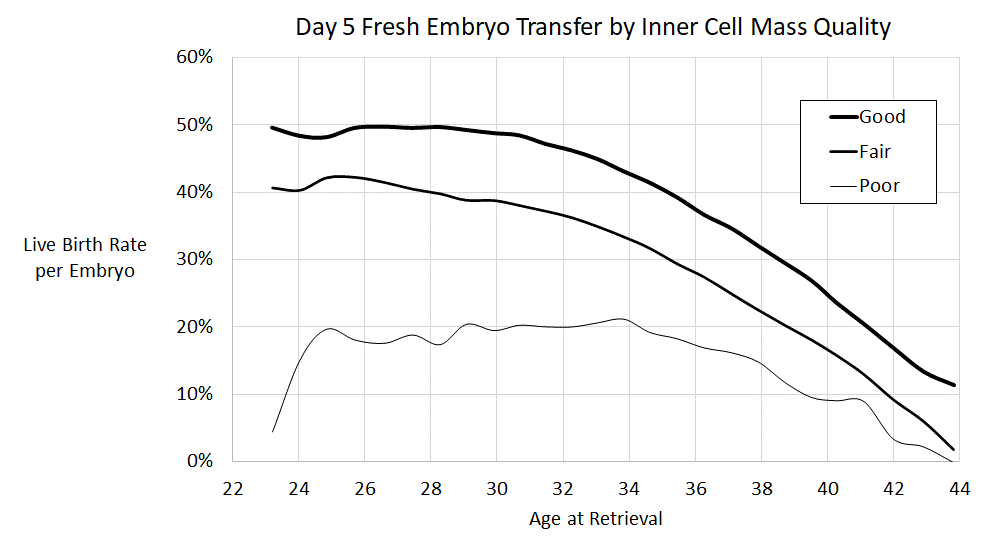


## Supplemental Figure 9


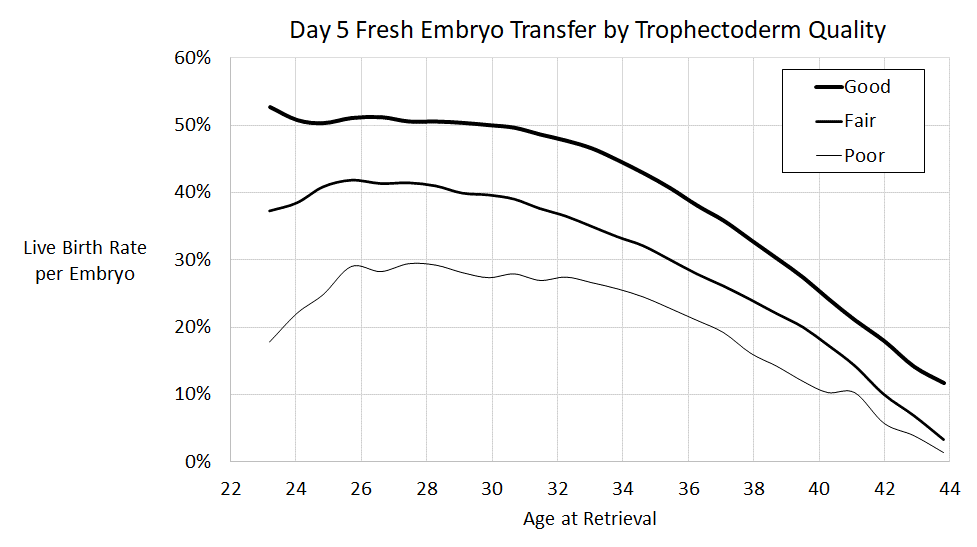


## Supplemental Figure 10


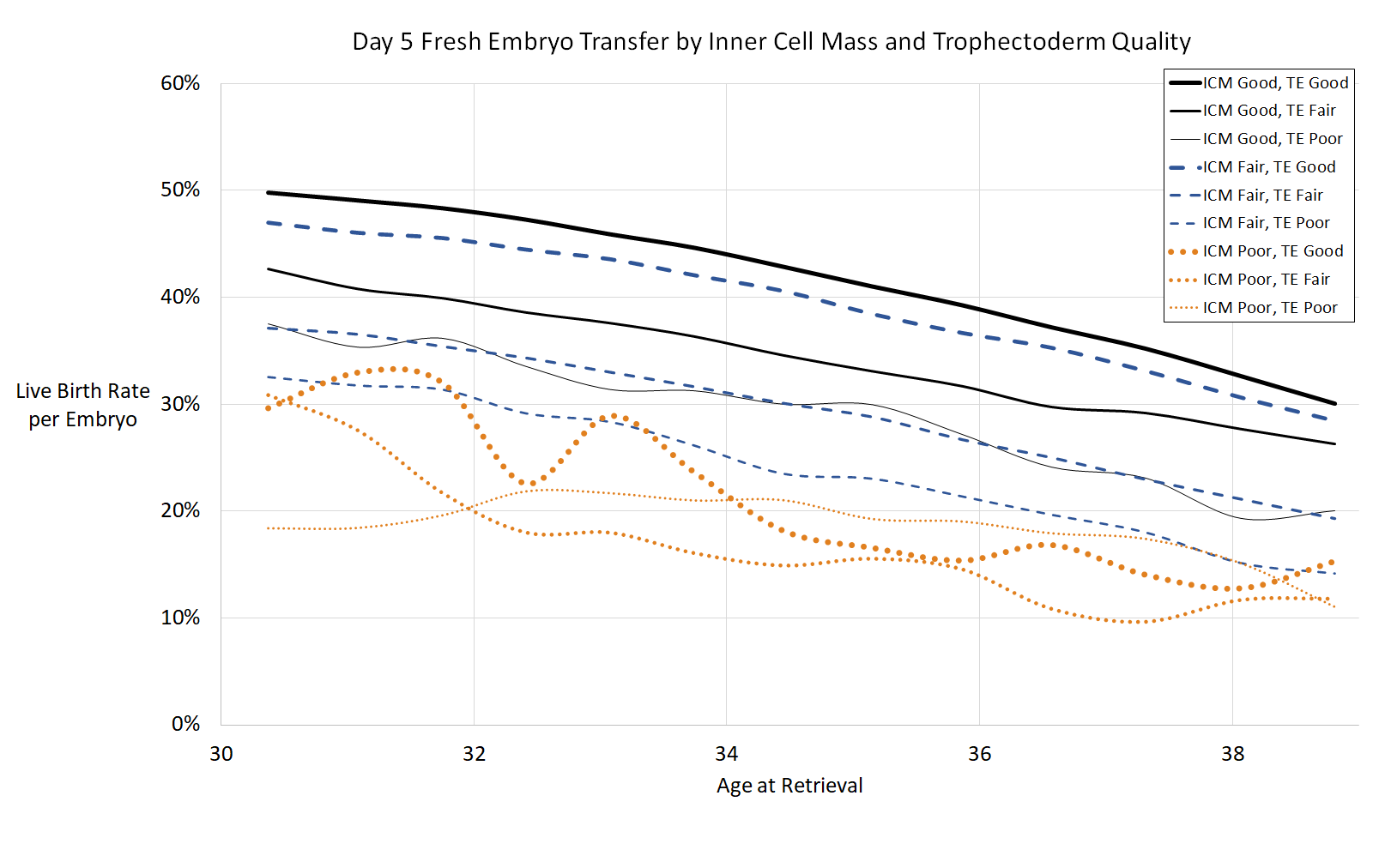


## Supplemental Figure 11


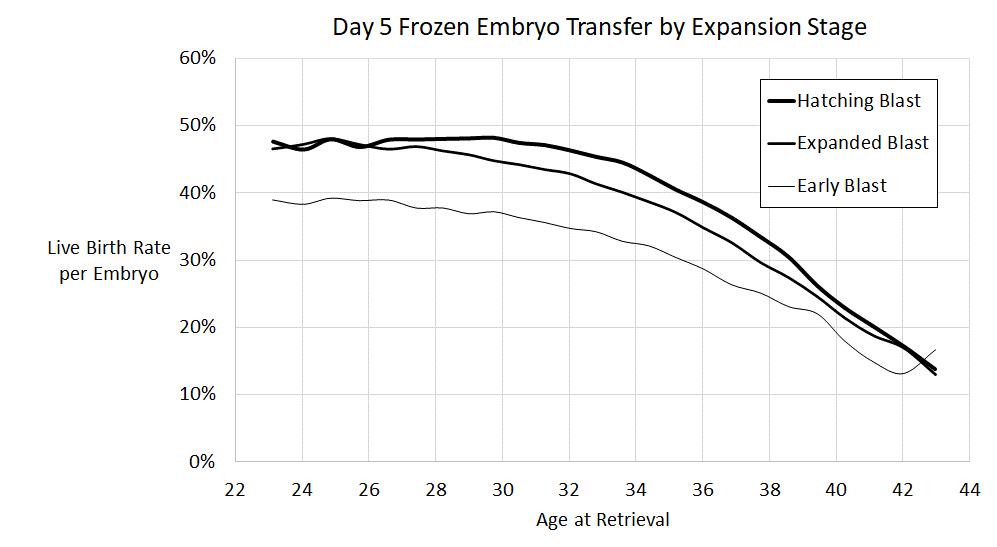


## Supplemental Figure 12


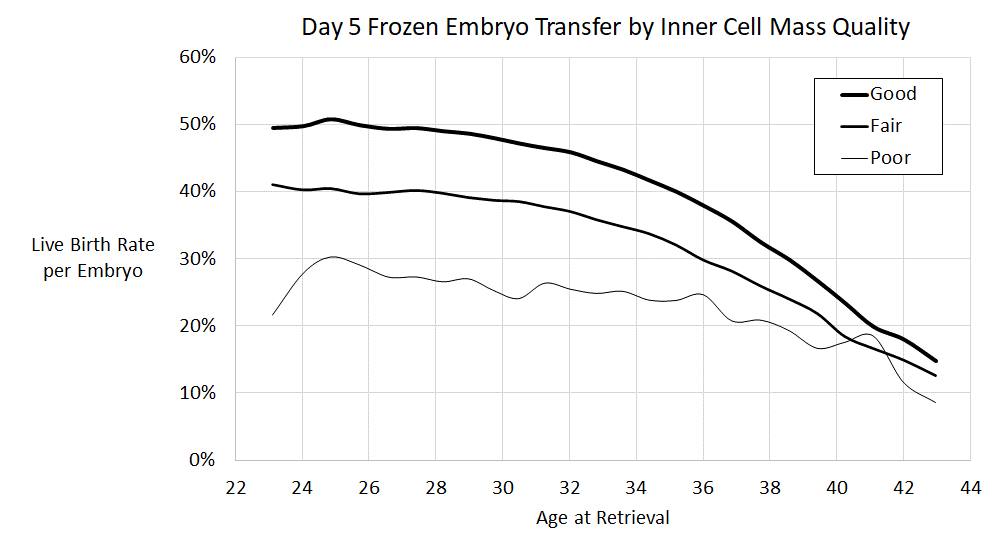


## Supplemental Figure 13


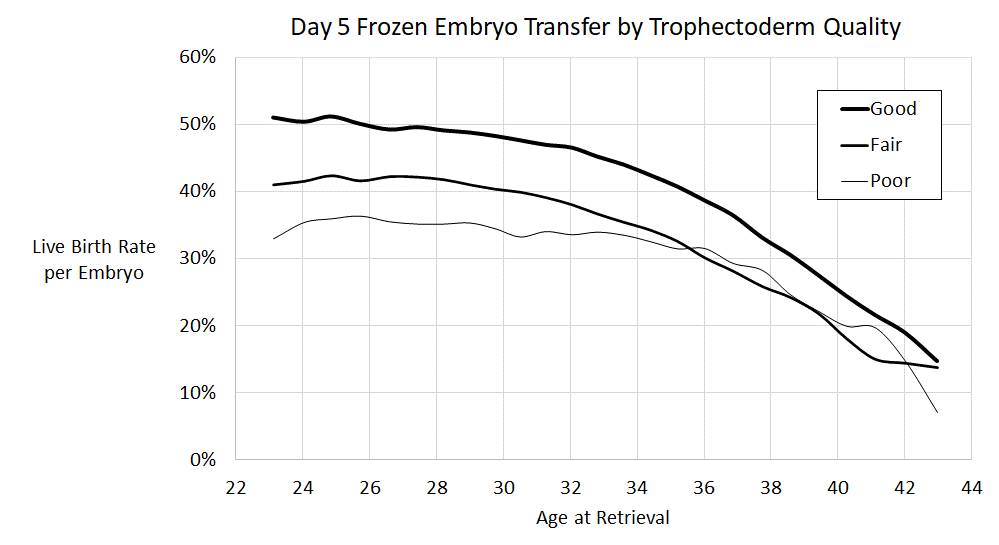


## Supplemental Figure 14


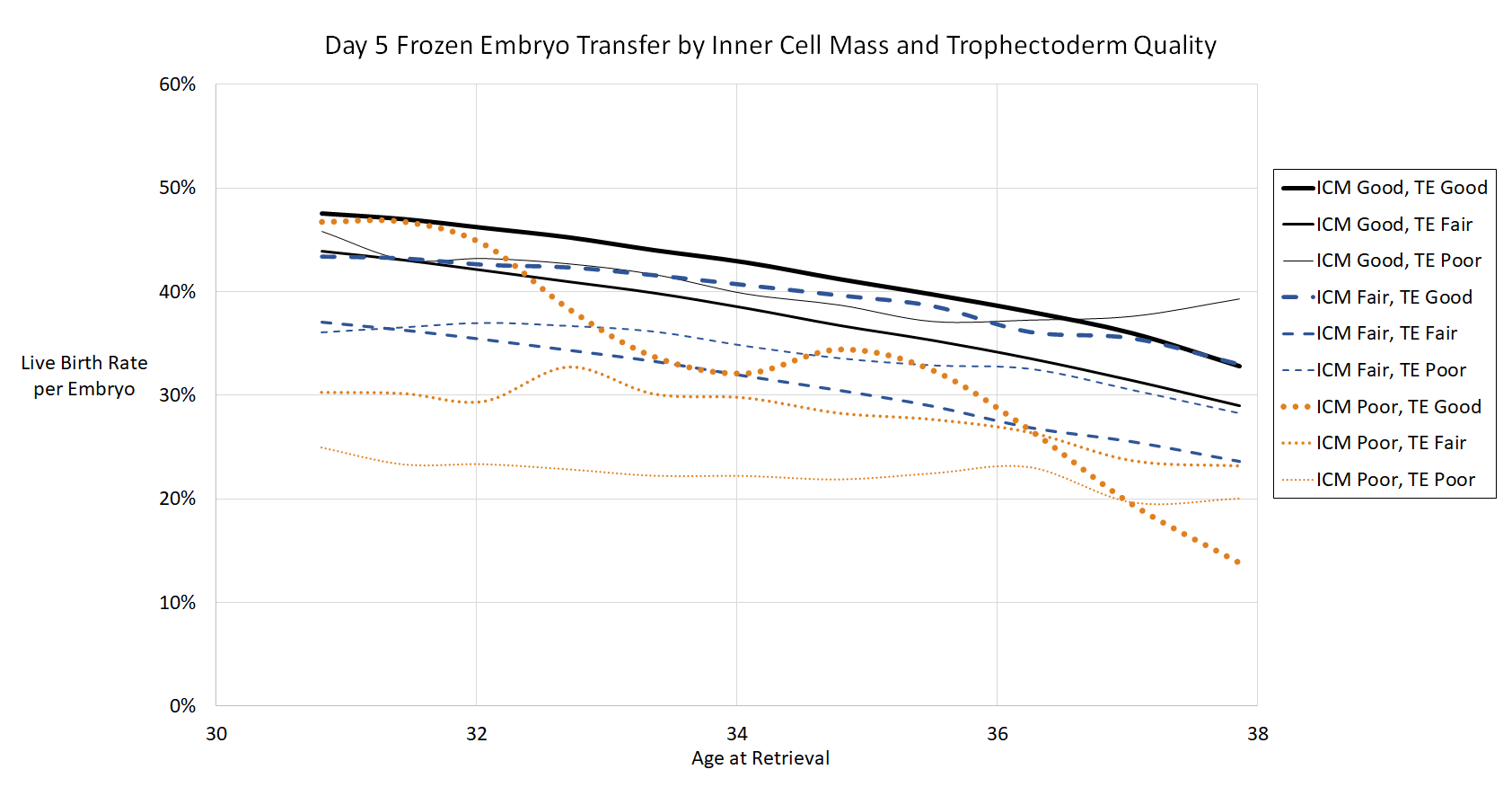


## Supplemental Figure 15


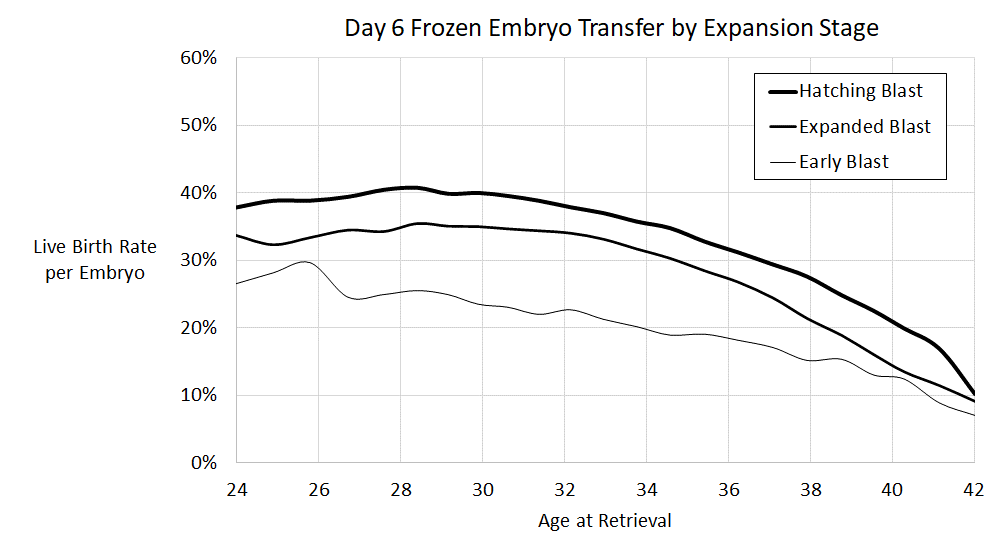


## Supplemental Figure 16


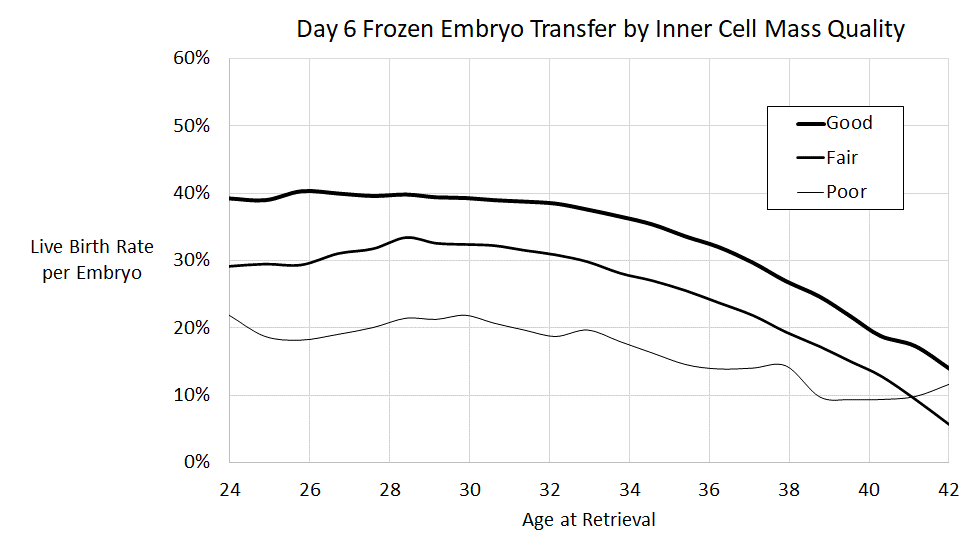


## Supplemental Figure 17


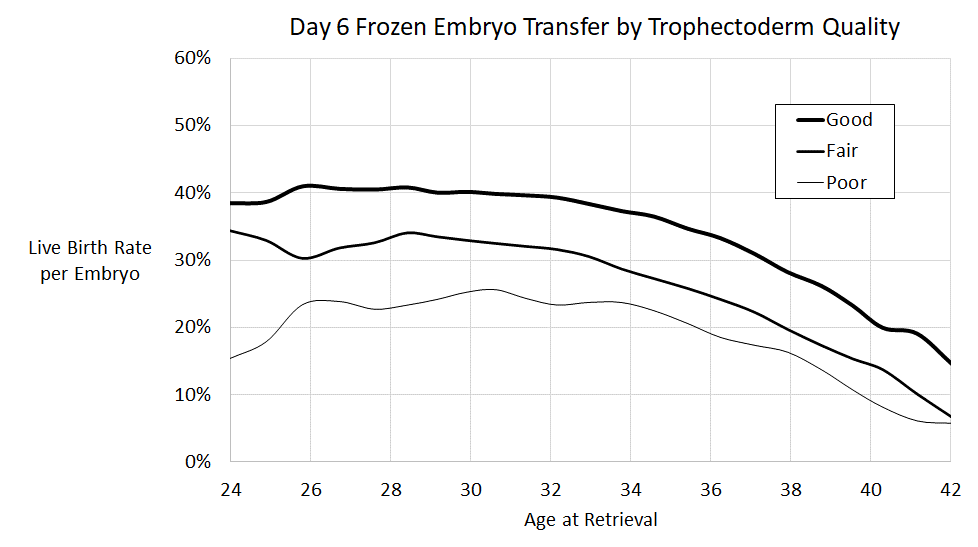


## Supplemental Figure 18


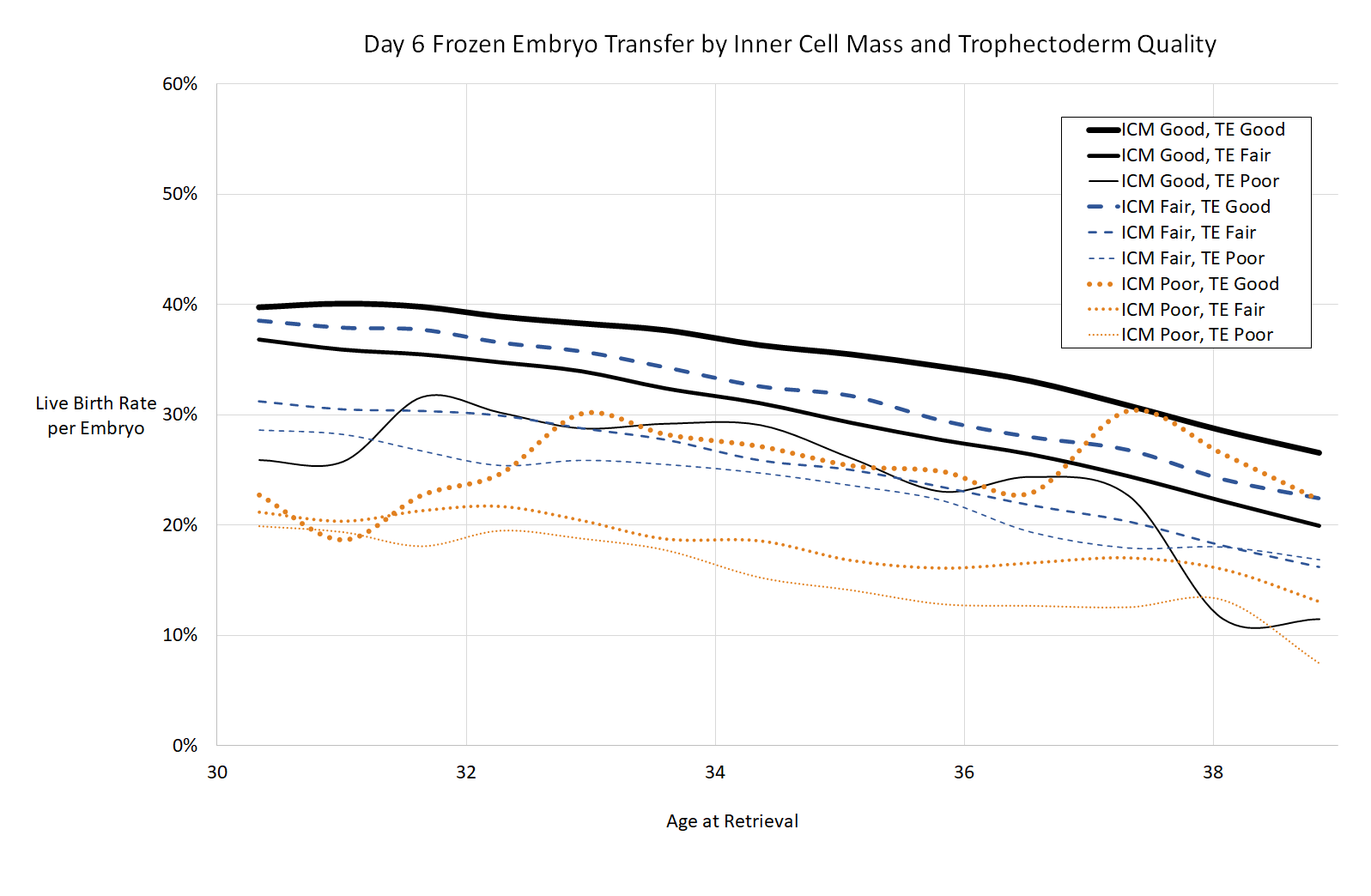


**Supplemental Figure 19**


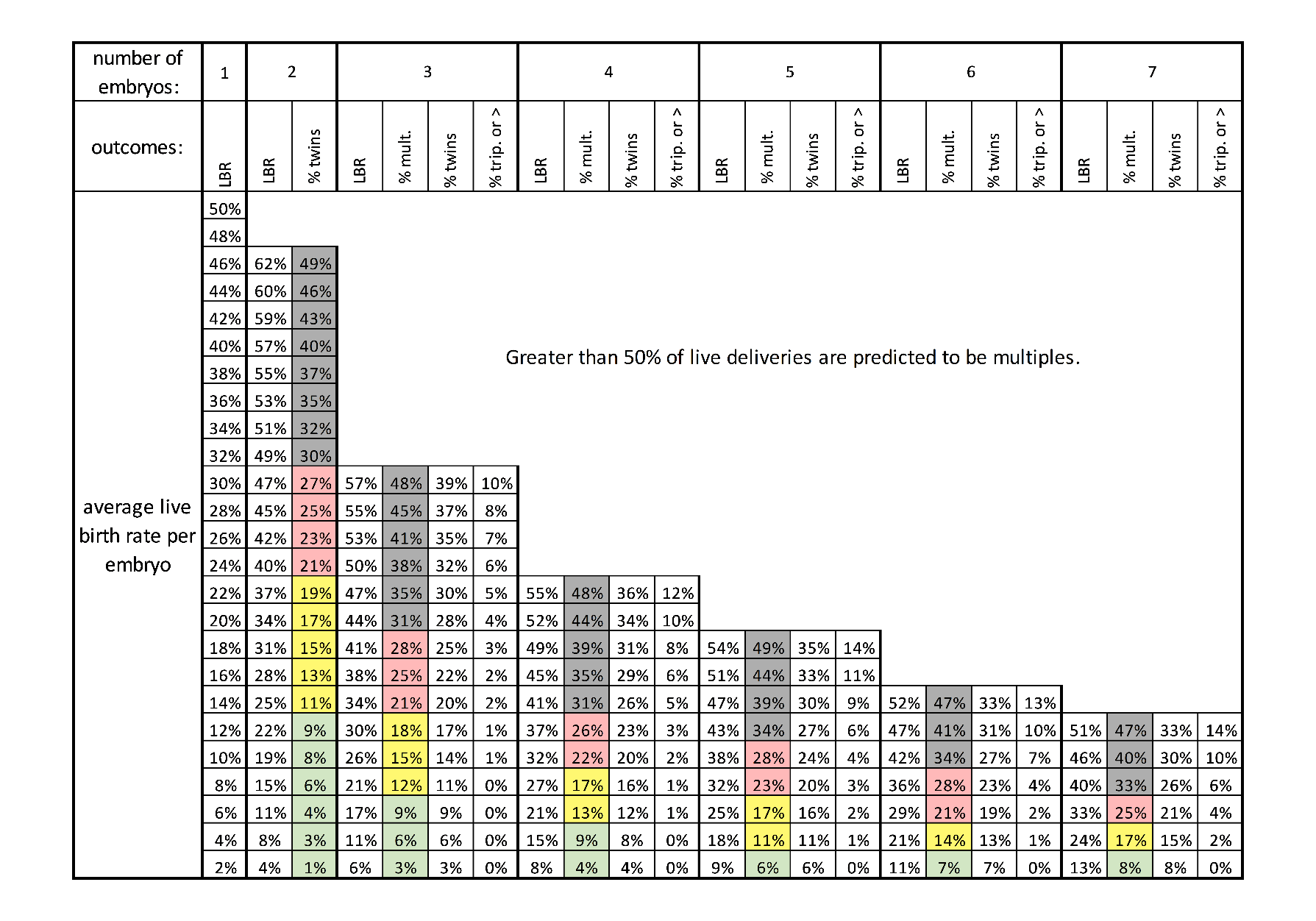


## Supplemental Figure 20


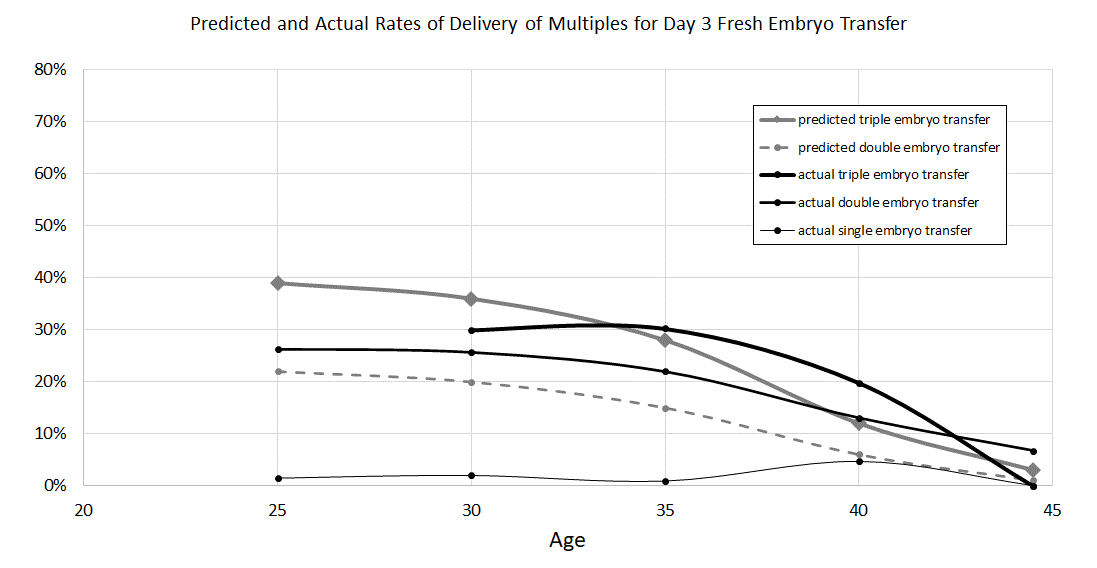


**Supplemental Figure 21**
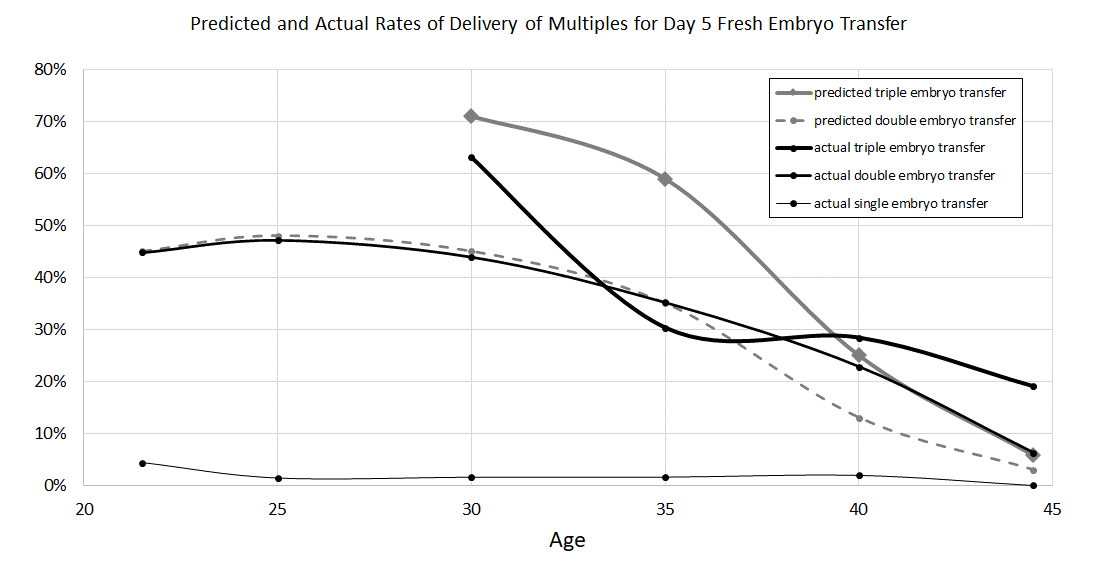


## Supplemental Figure 22


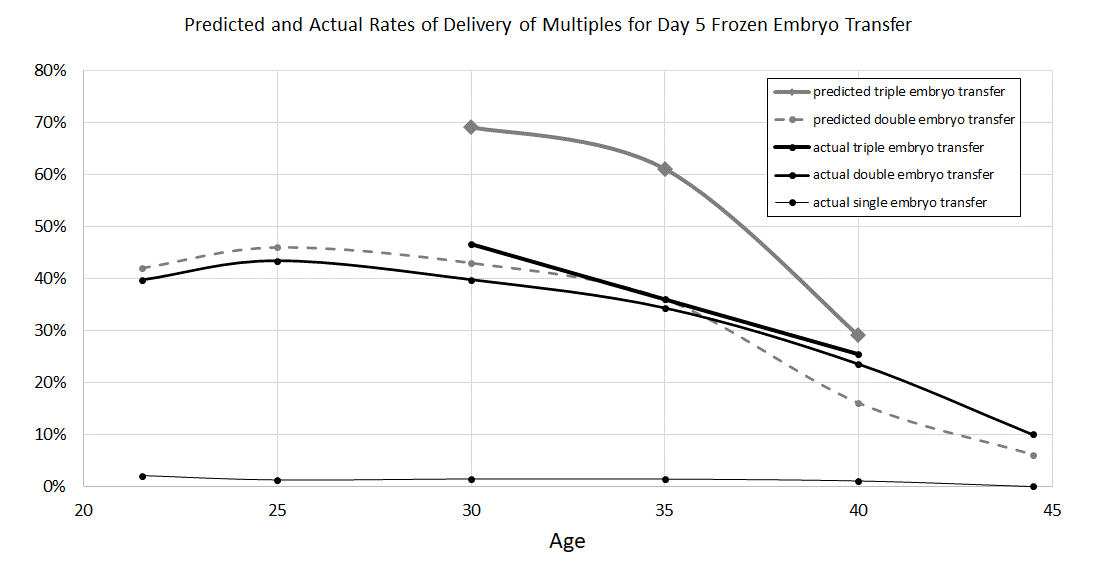


## Supplemental Figure 23


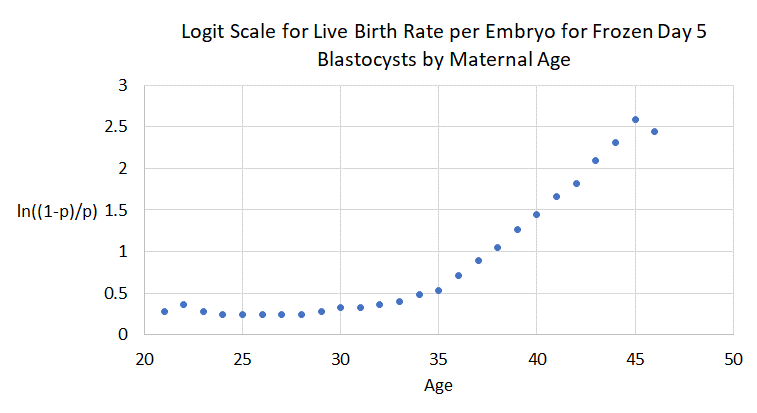

Supplement: Supplemental Figures [file mmc1.docx]
