# Supplementary material for: Embryo morphology and live birth in the United States
Source: F S Rep. 2022 Feb 23;3(2):131–7. doi: 10.1016/j.xfre.2022.02.006 (PMC9250116; doi:10.1016/j.xfre.2022.02.006)
Supplement: Supplemental Methods and Discussion [file mmc2.docx]

**Supplemental Methods and Discussion**

Embryo Morphology and Live Birth in the United States

Michael S. Awadalla, M.D., Jacqueline R. Ho, M.D., Lynda K. McGinnis, Ph.D., Ali Ahmady, Ph.D., Victoria K. Cortessis, Ph.D., Richard J. Paulson, M.D.

Table of Contents

[Supplemental Methods 1](#_Toc95983048)

[Study Population 1](#_Toc95983049)

[Linear Algebra Model and Moving Age Groups 1](#_Toc95983050)

[Statistical Methods 2](#_Toc95983051)

[Live Birth Rate per Embryo by Maternal Age, Embryo Stage, and Fresh/Frozen Transfer 3](#_Toc95983052)

[Single Versus Multiple Embryo Transfer 3](#_Toc95983053)

[Day 3 Cleavage Stage Detailed Morphology Analysis 3](#_Toc95983054)

[Day 5 and 6 Blastocyst Detailed Morphology Analysis 3](#_Toc95983055)

[Supplemental Discussion 4](#_Toc95983056)

[Implication of Results for Logistic Regression Modeling 4](#_Toc95983057)

[Day 6 Embryo Transfer Data Should be Interpreted With Caution 4](#_Toc95983058)

[Frozen Day 5 Blastocyst Transfer May be Better Than Fresh Transfer at Older Ages 4](#_Toc95983059)

[References 5](#_Toc95983060)

# Supplemental Methods

## Study Population

The SART CORS database contains comprehensive data from >90% of all clinics performing ART cycles in the United States. The data were collected through voluntary submission, verified by SART, and then reported to the Centers for Disease Control and Prevention in compliance with the Fertility Clinic Success Rate and Certification Act of 1992 (Public Law 102-493). SART maintains HIPAA-compliant business associates agreements with reporting clinics. In 2004, following a contract change with CDC, SART gained access to the SART CORS data system for the purposes of conducting research. The data in the SART CORS are validated annually with select clinics having on-site visits for chart review based on an algorithm for clinic selection(1).  During each visit, data reported by the clinic were verified with information recorded in patients’ charts(1).  In 2012, records for 2,045 cycles at 35 clinics were randomly selected for full validation, along with 238 egg or embryo banking cycles. The full validation included review of 1,318 cycles for which a pregnancy was reported. Among the non-donor cycles, 331 were multiple-fetus pregnancies. Ten out of 11 data fields selected for validation were found to have discrepancy rates of ≤5%. The exception was the diagnosis field, which, depending on the diagnosis, had a discrepancy rate between 2.1% and 9.2%(1).

In this study there were 39,860 day 3 cleavage stage transfers with 81,871 embryos for an average of 2.05 embryos per transfer. There were 183,517 blastocyst transfers with 255,017 embryos for an average of 1.39 embryos per transfer.

## Linear Algebra Model and Moving Age Groups

We have previously described use of linear algebra and moving age groups for analysis of single clinic data to determine best fit live birth rates per embryo based on cleavage and blastocyst stage embryo morphology(2,3). The same methods apply to multicenter data. Briefly, we use linear algebra to model embryo transfers. If we consider transfer of two good quality embryos resulting in one live birth the best fit live birth rate would be 50% per embryo. If we add in a second transfer of one good quality embryo and two fair quality embryos resulting in one live birth then the best fit live birth rate for the good quality embryos would still be 50% per embryo and the best fit live birth rate of each fair quality embryo would be 25%. With these percentages adding the live birth rates of each embryo transferred perfectly adds up to the number of babies born. Any number of embryo quality categories can be considered. When modeling more than a few embryo transfers there are no perfect solutions so linear algebra is used to give the best fit (least-squares) solution. We use MATLAB to perform the linear algebra which is capable of solving hundreds of thousands of equations in under a second.

We use moving age groups to smooth the data. This is similar to using a moving average. For each group of embryo data (such as day 5 fresh blastocyst transfers) we repeat the analysis of live birth rates five times at each integer of age. First, we consider only that exact maternal age expressed as an integer such as 34 years old. Then we repeat the analysis for 34 ± 1 year, 34 ± 2 years, 34 ± 3 years, and 34 ± 4 years. This corresponds to 1-year, 3-year, 5-year, 7-year, and 9-year moving groups. We then look at all the live birth rates for each embryo quality across all age groups. We expect from previous studies and biological plausibility that the live birth rate for an embryo has a smooth transition across ages and decreases as age increases(2,3). If there is random variation in the live birth rate as age increases more smoothing is needed by choosing a larger size moving group such as 5, 7, or 9-year moving groups. The more categories of embryo quality considered the larger the age group needed to provide adequate smoothing to interpret the data. At the extremes of age where there is less data overfitting is observed and this data is omitted. If embryo quality is broken down into more groups, we need to use a larger size age group or consider a narrower age range. The embryo morphologies are broken down into as many clinically relevant groups as is reasonable based on the dataset by considering the morphology distribution of the data. The live birth rates are graphed at the mean age for transfers in that group since this may be slightly different than the original mean age.

## Statistical Methods

Due to the large sample size, confidence in the reported live birth rates is limited more by methodology than sample size. For example, in the analysis of morphology overall grade for fresh day 5 blastocysts there were 52,045 included embryo transfers (Figure 1). In the 7-year age group centered on the mean age of 34 there were 31,001 transfers including 41,368 embryos (Supplemental Table 3). The fresh day 5 blastocysts were given overall grades of good 76% of the time, fair 21% of the time, and poor 3% of the time. For poor quality embryos our confidence interval on the live birth rate would be similar to that of approximately 930 (31,001 x 0.03) single poor quality embryo transfers which would give us a live birth rate of 21% with 95% CI 18-24%. The live birth rate for good quality fresh day 5 blastocysts is 43% with a CI of 42.4% to 43.6%. We have discussed methodology for determining confidence intervals on live birth rates determined with linear algebra(3) but this is of little value with large datasets compared to the complexity it adds. Some sense of the confidence in the data at the extremes of age can be seen by the amount of variation in the data. Most of the live birth rates around our mean age of 34 in parts of the analysis with lots of data are almost completely smooth while there is some random variation seen at the extremes of age.

There are several other limitations of these statistical methods. Generalizability of these results may be limited to the kinds of clinics that report to SART because clinics reporting data to SART may have different LBRs per embryo than clinics that do not. There is likely some residual confounding of embryo quality on reported live birth rates. Typically, the best embryos are selected for fresh transfer while remaining embryos are cryopreserved. At younger ages there may be more frequent use of embryo cryopreservation to reduce the risk of ovarian hyperstimulation syndrome and because of higher numbers of embryos from each cycle. At older ages there may be more use of fresh embryo transfer since there may only be a few embryos and fresh transfer avoids additional costs associated with embryo cryopreservation. Because there is no universal embryo grading system the results are likely to reflect some degree of information bias. Presuming that centers use the same classification for all patients, inconsistencies in coding are expected to be non-differential with respect to maternal age and live birth; thus, true differences in LBR between embryo quality groups may be somewhat greater than those reported. Day 3 cleavage stage embryo grading is more standardized than blastocyst grading because embryo cell number is universally recorded on day 3. Although the SART CORS does have standardized grading of embryo morphology there is still a substantial amount of subjectivity especially with assigning overall blastocyst morphology grades of good, fair, and poor(4).

## Live Birth Rate per Embryo by Maternal Age, Embryo Stage, and Fresh/Frozen Transfer

For day 3 cleavage stage morphology the most objective measurement of embryo morphology is the cell number on day 3. Embryo cell fragmentation and cell symmetry are other quantitative but more subjective morphology characteristics commonly assessed. Although other more specific methods of day 3 morphology assessment have been used(5–7), we prefer to focus on maternal age and day 3 cell number as these are universally recorded measures. In the 8-cell fresh cleavage stage embryo group there is enough data to break the analysis into groups based on fragmentation percentage. We prefer to focus on fragmentation percentage rather than cell symmetry because we feel that this is more objective and it is necessary to pick one or the other to include because we have found collinearity between the two in analysis of our own clinic data(2). The standardized grading used by SART includes cell number (1, 2, 3, 4, 5, 6, 7, 8, or > 8), fragmentation (0%, 1-10%, 11-25%, or >25%), and symmetry (perfect, moderately asymmetric, or severely asymmetric).

The most commonly referenced publication on blastocyst morphology is a 1999 publication by Gardner and Schoolcraft(8). Blastocyst expansion is categorized from 1 to 6 based on increasing degree of expansion (1 for early blastocyst, 2 for blastocyst, 3 for full blastocyst, 4 for expanded blastocyst, 5 for hatching blastocyst, and 6 for hatched blastocyst). ICM is graded as A for tightly packed with many cells, B for loosely grouped with several cells, or C for very few cells. TE is graded as A for many cells forming a cohesive epithelium, B for few cells forming loose epithelium, or C for very few large cells. The standardized grading used by SART is similar to that proposed by Gardner and Schoolcraft but is not specifically using that system(4,8). SART allows clinics to given an overall blastocyst grade of good, fair or poor but does not specify further how clinics must assign these grades. Expansion stage is specified as early, expanded, or hatching blastocyst. ICM and TE are each given scores of good, fair, or poor. Individual clinics can use their own methods to determine how their embryo data is entered into SART. Many clinics enter the same grade (good, fair, or poor) for overall grade, ICM grade, and TE grade. Other clinics use other methods in varying degrees of detail. Some clinics may not pay much attention to this part of the data entry at all. Other clinics that use the system presented by Gardner and Schoolcraft likely use a system of A = good, B = fair, and C = poor for ICM and TE grading and assign overall grade based on their own protocol.

## Single Versus Multiple Embryo Transfer

We performed analysis of live birth rates per embryo for day 3 and day 5 embryos by grouping the embryos in to groups of single or multiple embryo transfer to evaluate if embryos transferred in single embryo transfers had higher live birth rates than embryos transferred in multiple embryo transfers. For day 3 fresh transfers there were 32,870 transfers including 9,160 single embryo transfers (SET) and 23,710 multiple embryo transfers (MET). For day 3 frozen transfers there were 6,990 transfers including 2,125 SET and 4,865 MET. For day 5 fresh transfers there were 63,668 transfers including 37,288 SET and 26,380 MET. For day 5 frozen transfers there were 80,041 transfers including 52,038 SET and 28,003 MET.

## Day 3 Cleavage Stage Detailed Morphology Analysis

There were 39,860 day 3 embryo transfers (32,870 fresh and 6,990 frozen transfers). We removed 2,847 cycles with transfer of more than 3 embryos. 2,869 transfers including embryos with a stage listed as having fewer than 4 cells or blastocyst stage were removed as these categories contained low numbers of transfers and including them would risk overfitting the data. 16 transfer cycles with a maternal age greater than 49 years were excluded. Morphology of the remaining 34,128 day 3 cleavage stage transfers (28,878 fresh transfers and 5,250 frozen transfers) was analyzed by maternal age and cell number. Based on the morphology distribution (Supplemental Fig. 5) there was enough data to break up the 8-cell group into 3 additional groups based on fragmentation percentage for fresh transfers. There were 26,672 fresh day 3 transfers with cell fragmentation data available. Best fit live birth rates based on cleavage stage morphology are given in Figure 4.

## Day 5 and 6 Blastocyst Detailed Morphology Analysis

There were 63,668 fresh day 5 embryo transfers. To simplify the morphology analysis, we removed 1,601 transfers with more than 2 embryos. 10,022 transfers were removed due to no data for overall morphology grade, expansion, inner cell mass quality, or trophectoderm quality. The remaining 52,045 embryo transfers included 71,418 embryos and resulted in 28,167 live born fetuses for a 39.4% live birth rate per embryo. Best fit rates of live birth are given for 5 subgroups: overall grade (good, fair, or poor), expansion stage (early blast, expanded blast, or hatching blast), ICM grade (good, fair, or poor), TE grade (good, fair, or poor), and all 9 combinations of ICM and TE grade). There was moderate agreement of the ICM and TE grade of fresh day 5 embryos as the Cohen’s kappa statistic was 0.50.

There were 80,041 frozen day 5 embryo transfers. There were 931 transfers of greater than 2 embryos removed and 22,219 transfers removed with no data for one or more groups of embryo morphology. The remaining 56,891 embryo transfers included 75,719 embryos and resulted in 31,495 live born fetuses for a 41.6% live birth rate. Best fit live birth rates are given for 5 subgroups. There was moderate agreement of the ICM and TE grade of frozen day 5 embryos as the Cohen’s kappa statistic was 0.52.

There were 36,404 frozen day 6 embryo transfers. 494 transfers of greater than 2 embryos were removed and 12,632 transfers were removed for no data for one or more morphology group. The remaining 23,278 embryo transfers included 31,095 embryos and resulted in 9,976 live born fetuses for a 32.1% live birth rate per embryo. There was not enough data to perform morphology analysis for fresh day 6 embryos or day 7 embryos.

# Supplemental Discussion

## Implication of Results for Logistic Regression Modeling

Logistic regression is the most common method of evaluating embryo morphology and live birth rates in published literature. We found live birth rate per embryo to fit the logit assumption for ages 35 to 45 years but not across all ages from 20 to 45 years (Supplemental Fig. 23). The logit assumption necessary for logistic regression would not have been met with this dataset. This suggests that studies using logistic regression to model birth rates should be interpreted with caution and with attention to how the assumptions of logistic regression were evaluated. From age 21 through 34 there was a similar live birth rate per embryo in most of our analysis with only a slight decrease as in live birth rates as age approached 34 years. This is best seen in Figure 2A. This finding supports current SART practice of reporting outcomes for <35 years as one age group (the other age groups are 35-37, 38-40, 41-42, and > 42). Use of logistic regression to model embryo transfer and IVF outcomes should not be performed with data from maternal ages both below and above 35 years unless the logit assumption is assessed in the dataset. ICM and TE quality should not both be used in the same model due to significant collinearity (Supplemental Fig. 6).

Our data suggests logistic regression could only be used for maternal ages 35 and over. Data from ages 21 to 34 could likely be analyzed without controlling for age in many datasets such as ours where live birth rates are similar across all ages in this group. To include data from all ages and use logistic regression an age value of 34 years could be entered into the logistic regression model for all ages from 21 to 34 years.

## Day 6 Embryo Transfer Data Should be Interpreted With Caution

Day 6 fresh and frozen data are challenging to interpret. For day 6 fresh transfers there may be significant asynchrony between the embryo and endometrium since progesterone elevation prior to oocyte retrieval is common in fresh cycles. Some centers perform day 6 embryo transfer for retrievals that occur later in the day and this may not have as much asynchrony as embryos form retrievals performed early in the day with fresh transfer on day 6. The reasons for day 6 and day 7 fresh transfer may vary from clinic to clinic. Some clinics may have embryology staffing constraints on the weekends.

Day 6 frozen embryo transfer data are also difficult to interpret since the optimal duration of progesterone in frozen cycles has not yet been determined. Most clinics transfer frozen day 6 embryos on the same timing as day 5 embryos (approximately 108-120 hours after the start of progesterone exposure). However, one retrospective cohort study found higher live birth rates for day 6 embryos transferred after 7 compared to 6 days of progesterone (36% vs 22%, p = 0.06)(9). This suggests an additional day of progesterone prior to frozen day 6 embryo transfer may be optimal.

## Frozen Day 5 Blastocyst Transfer May be Better Than Fresh Transfer at Older Ages

We have observed for the first time that at ages 40 and above the live birth rate per embryo is 2-4% higher in day 5 frozen embryo transfers than day 5 fresh embryo transfers (Figure 2A). This effect persisted when controlling for embryo overall grade (Figure 3A&B). This is also seen in our own clinic data however we did not have enough data to control for embryo overall grade(10). This association suggests that a prospective randomized controlled trial should be performed including women 40 years and over (or perhaps including all women 35 and over) based on intention to treat with fresh or frozen embryo transfer.

It cannot be concluded that frozen embryo transfer is preferrable in these patients because selection bias from retrospective data could bias results in favor of fresh or frozen transfer. Even when controlling for embryo grade there may be more low-quality embryos transferred in fresh cycles. In routine practice low quality embryos are often transferred in fresh cycles rather than being discarded even if they do not meet criteria for cryopreservation. This would artificially make fresh transfer seem worse than frozen transfer. On the other hand, when there are surplus embryos beyond a safe number that can be transferred at once the best embryos are always transferred fresh and the lower quality embryos cryopreserved. Although LBRs are similar for fresh and frozen embryos in younger patients and different in older patients, this may be a result of more selection bias for embryo transfers in older patients.

# References

1. Center for Disease Control and Prevention, American Society for Reproductive Medicine, and Society for Assisted Reproductive Technology. 2012 Assisted Reproductive Technology and Success Rates: National Summary and Fertility Clinic Reports. Washington, DC; 2014

2. Awadalla M, Vestal N, McGinnis L, Ahmady A. Effect of Age and Morphology on Live Birth Rate After Cleavage Stage Embryo Transfer. Reprod Sci 2021;43–51. https://doi.org/10.1007/s43032-020-00249-9

3. Awadalla M, Kim A, Vestal N, Ho J, Bendikson K. Effect of Age and Embryo Morphology on Live Birth Rate After Transfer of Unbiopsied Blastocysts. JBRA Assist Reprod 2020;https://doi.org/10.5935/1518-0557.20200101

4. Racowsky C, Vernon M, Mayer J, Ball GD, Behr B, Pomeroy KO, et al. Standardization of grading embryo morphology. J Assist Reprod Genet 2010;27:437–9. https://doi.org/10.1007/s10815-010-9443-2

5. Fisch JD, Sher G, Adamowicz M, Keskintepe L. The graduated embryo score predicts the outcome of assisted reproductive technologies better than a single day 3 evaluation and achieves results associated with blastocyst transfer from day 3 embryo transfer. https://doi.org/10.1016/j.fertnstert.2003.05.013

6. Racowsky C, Ohno-Machado L, Kim J, Biggers JD. Is there an advantage in scoring early embryos on more than one day? Hum Reprod 2009;24:2104–13. https://doi.org/10.1093/humrep/dep198

7. Chen C, Kattera S. Comparison of pronuclear zygote morphology and early cleavage status of zygotes as additional criteria in the selection of day 3 embryos: A randomized study. Fertil Steril 2006;85:347–52. https://doi.org/10.1016/j.fertnstert.2005.07.1319

8. Gardner DK, Schoolcraft WB. In vitro culture of human blastocysts. In: Jansen R, Mortimer D, eds. Towards Reproductive Certainty: Fertility and Genetics beyond. 1999;378–88.

9. Roelens C, Santos-Ribeiro S, Becu L, Mackens S, Van Landuyt L, Racca A, et al. Frozen-warmed blastocyst transfer after 6 or 7 days of progesterone administration: impact on live birth rate in hormone replacement therapy cycles. Fertil Steril 2020;114:125–32. https://doi.org/10.1016/j.fertnstert.2020.03.017

10. Awadalla MS, Bendikson KA, Ho JR, McGinnis LK, Ahmady A. A validated model for predicting live birth after embryo transfer. Sci Rep 2021;11:1–8. https://doi.org/10.1038/s41598-021-90254-y
