# Supplementary material for: Embryo morphology and live birth in the United States
Source: F S Rep. 2022 Feb 23;3(2):131–7. doi: 10.1016/j.xfre.2022.02.006 (PMC9250116; doi:10.1016/j.xfre.2022.02.006)
Supplement: Supplemental Tables [file mmc3.docx]

**Supplemental Tables**

Embryo Morphology and Live Birth in the United States

Michael S. Awadalla, M.D., Jacqueline R. Ho, M.D., Lynda K. McGinnis, Ph.D., Ali Ahmady, Ph.D., Victoria K. Cortessis, Ph.D., Richard J. Paulson, M.D.

Table of Contents

[Supplemental Table 1: Demographics and cycle characteristics. 2](#_Toc95996175)

[Supplemental Table 2: Best fit live birth rate per embryo based on maternal age at oocyte retrieval, embryo day, and fresh or frozen embryo transfer. 2](#_Toc95996176)

[Supplemental Table 3: Best fit live birth rates based on day 5 fresh embryo morphology overall grade. 3](#_Toc95996177)

[Supplemental Table 4: Best fit live birth rates based on day 5 fresh embryo morphology expansion stage. 3](#_Toc95996178)

[Supplemental Table 5: Best fit live birth rates based on day 5 fresh embryo morphology inner cell mass quality. 3](#_Toc95996179)

[Supplemental Table 6: Best fit live birth rates based on day 5 fresh embryo morphology trophectoderm quality. 3](#_Toc95996180)

[Supplemental Table 7: Best fit live birth rates based on day 5 fresh embryo morphology inner cell mass and trophectoderm quality. 3](#_Toc95996181)

[Supplemental Table 8: Best fit live birth rates based on day 5 frozen embryo morphology overall grade. 4](#_Toc95996182)

[Supplemental Table 9: Best fit live birth rates based on day 5 frozen embryo morphology expansion stage. 4](#_Toc95996183)

[Supplemental Table 10: Best fit live birth rates based on day 5 frozen embryo morphology inner cell mass quality. 4](#_Toc95996184)

[Supplemental Table 11: Best fit live birth rates based on day 5 frozen embryo morphology trophectoderm quality. 4](#_Toc95996185)

[Supplemental Table 12: Best fit live birth rates based on day 5 frozen embryo morphology inner cell mass and trophectoderm quality. 4](#_Toc95996186)

[Supplemental Table 13: Best fit live birth rates based on day 6 frozen embryo morphology overall grade. 5](#_Toc95996187)

[Supplemental Table 14: Best fit live birth rates based on day 6 frozen embryo morphology expansion stage. 5](#_Toc95996188)

[Supplemental Table 15: Best fit live birth rates based on day 6 frozen embryo morphology inner cell mass quality. 5](#_Toc95996189)

[Supplemental Table 16: Best fit live birth rates based on day 6 frozen embryo morphology trophectoderm quality. 5](#_Toc95996190)

[Supplemental Table 17: Best fit live birth rates based on day 6 frozen embryo morphology inner cell mass and trophectoderm quality. 5](#_Toc95996191)

[Supplemental Table 18: Best fit live birth rates based on day 3 cleavage stage embryo morphology for fresh embryo transfers. 7](#_Toc95996192)

[Supplemental Table 19: Best fit live birth rates based on day 3 cleavage stage embryo morphology for fresh transfers with analysis of 8-cell embryo fragmentation. 7](#_Toc95996193)

[Supplemental Table 20: Best fit live birth rates based on day 3 cleavage stage embryo morphology for frozen embryo transfers. 7](#_Toc95996194)

## Supplemental Table 1: Demographics and cycle characteristics.

| n = 237,160 | |
| --- | --- |
| **Age** (years) | 33.9 (4.5) |
| **BMI** (kg/m^2^) | 26.7 (6.7) |
| **Gravidity**  **Parity** | 1.2  0.5 |
| **Race/Ethnicity** | |
| White | 47.3 % |
| Asian | 9.4 % |
| African American | 5.7 % |
| Hispanic | 5.6 % |
| American Indian | 0.2 % |
| Native Hawaiian / Pacific | 0.2 % |
| Not asked / Refused / Unknown | 31.7 % |
| **Transfer Type (included cycles only) n = 223,377** | |
| Fresh day 3 cleavage | 14.7% |
| Frozen day 3 cleavage | 3.1% |
| Fresh day 5 blastocyst | 28.5% |
| Frozen day 5 blastocyst | 35.8% |
| Fresh day 6 blastocyst | 0.9% |
| Frozen day 6 blastocyst | 16.3% |
| Fresh day 7 blastocyst | 0.04% |
| Frozen day 7 blastocyst | 0.6% |
| **Stimulation Cycle (fresh cycles only) n = 98,625** | |
| GnRH Antagonist | 72.3 % |
| GnRH Agonist Suppression | 16.4 % |
| GnRH Flare | 11.3 % |

Data are given as mean, mean (SD), or percent.

## Supplemental Table 2: Best fit live birth rate per embryo based on maternal age at oocyte retrieval, embryo day, and fresh or frozen embryo transfer.


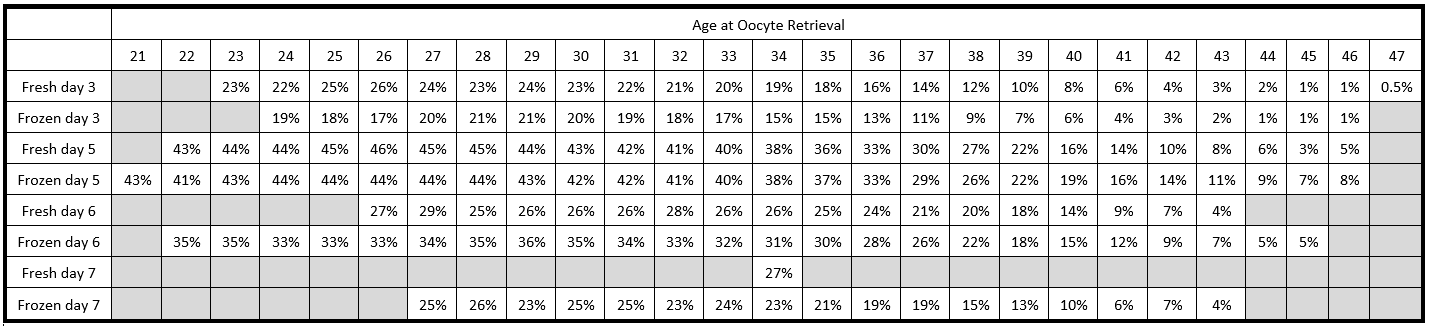


5-year moving age groups are used.

## Supplemental Table 3: Best fit live birth rates based on day 5 fresh embryo morphology overall grade.

|  | Best fit live birth rate per embryo | | | | | | | | | | | | | | | | | | | | | | | | | |
| --- | --- | --- | --- | --- | --- | --- | --- | --- | --- | --- | --- | --- | --- | --- | --- | --- | --- | --- | --- | --- | --- | --- | --- | --- | --- | --- |
| age | 23 | 24 | 25 | 26 | 27 | 27 | 28 | 29 | 30 | 31 | 31 | 32 | 33 | 34 | 35 | 35 | 36 | 37 | 38 | 39 | 40 | 40 | 41 | 42 | 43 | 44 |
| good | 49% | 49% | 49% | 50% | 50% | 50% | 50% | 49% | 49% | 49% | 48% | 46% | 45% | 43% | 42% | 39% | 37% | 34% | 32% | 29% | 26% | 23% | 20% | 16% | 13% | 11% |
| fair | 40% | 37% | 37% | 39% | 37% | 37% | 36% | 35% | 35% | 35% | 34% | 33% | 32% | 30% | 29% | 27% | 25% | 23% | 21% | 19% | 17% | 15% | 12% | 9% | 6% | 2% |
| poor | 15% | 20% | 19% | 25% | 22% | 24% | 25% | 24% | 23% | 23% | 22% | 22% | 22% | 21% | 20% | 19% | 18% | 16% | 14% | 12% | 9% | 8% | 7% | 2% | 0% | -1% |
| n transfers | 756 | 1408 | 2344 | 3854 | 6081 | 8980 | 12520 | 16379 | 20579 | 24586 | 28073 | 30423 | 31392 | 31001 | 29307 | 26553 | 23141 | 19044 | 14937 | 11274 | 8060 | 5546 | 3481 | 1955 | 1052 | 537 |
| n embryos | 980 | 1818 | 3028 | 4976 | 7851 | 11541 | 16118 | 21024 | 26369 | 31482 | 36048 | 39435 | 41213 | 41368 | 40068 | 37377 | 33661 | 28664 | 23205 | 18105 | 13316 | 9269 | 5823 | 3220 | 1698 | 842 |

52,045 embryo transfers included. 7-year moving age groups are used. N is given for 7-year age group centered on age of interest. Age groups with fewer than 500 transfers are omitted.

## Supplemental Table 4: Best fit live birth rates based on day 5 fresh embryo morphology expansion stage.

|  | Best fit live birth rate per embryo | | | | | | | | | | | | | | | | | | | | | | | | | |
| --- | --- | --- | --- | --- | --- | --- | --- | --- | --- | --- | --- | --- | --- | --- | --- | --- | --- | --- | --- | --- | --- | --- | --- | --- | --- | --- |
| age | 23 | 24 | 25 | 26 | 27 | 27 | 28 | 29 | 30 | 31 | 31 | 32 | 33 | 34 | 35 | 35 | 36 | 37 | 38 | 39 | 40 | 40 | 41 | 42 | 43 | 44 |
| early blast | 34% | 37% | 35% | 36% | 34% | 35% | 34% | 33% | 33% | 32% | 31% | 30% | 29% | 27% | 26% | 24% | 22% | 21% | 19% | 16% | 15% | 13% | 11% | 7% | 5% | 4% |
| expanded blast | 48% | 47% | 48% | 50% | 50% | 49% | 49% | 48% | 48% | 47% | 46% | 45% | 44% | 42% | 40% | 38% | 36% | 33% | 31% | 28% | 25% | 22% | 19% | 15% | 12% | 8% |
| hatching blast | 50% | 47% | 49% | 48% | 48% | 49% | 51% | 51% | 51% | 51% | 50% | 50% | 48% | 46% | 43% | 41% | 38% | 36% | 32% | 30% | 27% | 24% | 21% | 15% | 11% | 7% |
| n transfers | 756 | 1408 | 2344 | 3854 | 6081 | 8980 | 12520 | 16379 | 20579 | 24586 | 28074 | 30423 | 31392 | 31001 | 29307 | 26553 | 23141 | 19044 | 14937 | 11274 | 8060 | 5546 | 3481 | 1955 | 1052 | 537 |
| n embryos | 980 | 1818 | 3028 | 4976 | 7851 | 11541 | 16118 | 21024 | 26369 | 31482 | 36050 | 39435 | 41213 | 41368 | 40068 | 37377 | 33661 | 28664 | 23205 | 18105 | 13316 | 9269 | 5823 | 3220 | 1698 | 842 |

52,045 embryo transfers included. 7-year moving age groups are used. N is given for 7-year age group centered on age of interest. Age groups with fewer than 500 transfers are omitted.

## Supplemental Table 5: Best fit live birth rates based on day 5 fresh embryo morphology inner cell mass quality.

|  | Best fit live birth rate per embryo | | | | | | | | | | | | | | | | | | | | | | | | | |
| --- | --- | --- | --- | --- | --- | --- | --- | --- | --- | --- | --- | --- | --- | --- | --- | --- | --- | --- | --- | --- | --- | --- | --- | --- | --- | --- |
| age | 23 | 24 | 25 | 26 | 27 | 27 | 28 | 29 | 30 | 31 | 31 | 32 | 33 | 34 | 35 | 35 | 36 | 37 | 38 | 39 | 40 | 40 | 41 | 42 | 43 | 44 |
| good | 50% | 48% | 48% | 50% | 50% | 50% | 50% | 49% | 49% | 49% | 47% | 46% | 45% | 43% | 41% | 39% | 37% | 35% | 32% | 29% | 27% | 24% | 20% | 17% | 13% | 11% |
| fair | 41% | 40% | 42% | 42% | 41% | 40% | 40% | 39% | 39% | 38% | 37% | 36% | 35% | 33% | 32% | 29% | 27% | 25% | 22% | 20% | 18% | 16% | 13% | 9% | 6% | 2% |
| poor | 4% | 15% | 20% | 18% | 18% | 19% | 17% | 20% | 19% | 20% | 20% | 20% | 21% | 21% | 19% | 18% | 17% | 16% | 15% | 12% | 10% | 9% | 9% | 3% | 2% | 0% |
| n transfers | 756 | 1408 | 2344 | 3854 | 6081 | 8980 | 12520 | 16477 | 20579 | 24586 | 28074 | 30423 | 31392 | 31001 | 29307 | 26553 | 23141 | 19044 | 14937 | 11274 | 8060 | 5546 | 3481 | 1955 | 1052 | 537 |
| n embryos | 980 | 1818 | 3028 | 4976 | 7851 | 11541 | 16118 | 21139 | 26369 | 31482 | 36050 | 39435 | 41213 | 41368 | 40068 | 37377 | 33661 | 28664 | 23205 | 18105 | 13316 | 9269 | 5823 | 3220 | 1698 | 842 |

52,045 embryo transfers included. 7-year moving age groups are used. N is given for 7-year age group centered on age of interest. Age groups with fewer than 500 transfers are omitted.

## Supplemental Table 6: Best fit live birth rates based on day 5 fresh embryo morphology trophectoderm quality.

|  | Best fit live birth rate per embryo | | | | | | | | | | | | | | | | | | | | | | | | | |
| --- | --- | --- | --- | --- | --- | --- | --- | --- | --- | --- | --- | --- | --- | --- | --- | --- | --- | --- | --- | --- | --- | --- | --- | --- | --- | --- |
| age | 23 | 24 | 25 | 26 | 27 | 27 | 28 | 29 | 30 | 31 | 31 | 32 | 33 | 34 | 35 | 35 | 36 | 37 | 38 | 39 | 40 | 40 | 41 | 42 | 43 | 44 |
| good | 53% | 51% | 50% | 51% | 51% | 51% | 51% | 50% | 50% | 50% | 49% | 48% | 47% | 45% | 43% | 41% | 38% | 36% | 33% | 30% | 27% | 24% | 21% | 18% | 14% | 12% |
| fair | 37% | 38% | 41% | 42% | 41% | 41% | 41% | 40% | 40% | 39% | 38% | 36% | 35% | 33% | 32% | 30% | 28% | 26% | 24% | 22% | 20% | 17% | 14% | 10% | 7% | 3% |
| poor | 18% | 22% | 25% | 29% | 28% | 29% | 29% | 28% | 27% | 28% | 27% | 27% | 27% | 26% | 25% | 23% | 21% | 19% | 16% | 14% | 12% | 10% | 10% | 6% | 4% | 1% |
| n transfers | 756 | 1408 | 2344 | 3854 | 6081 | 8980 | 12520 | 16379 | 20579 | 24586 | 28074 | 30423 | 31392 | 31001 | 29307 | 26553 | 23141 | 19044 | 14937 | 11274 | 8060 | 5546 | 3481 | 1955 | 1052 | 537 |
| n embryos | 980 | 1818 | 3028 | 4976 | 7851 | 11541 | 16118 | 21024 | 26369 | 31482 | 36050 | 39435 | 41213 | 41368 | 40068 | 37377 | 33661 | 28664 | 23205 | 18105 | 13316 | 9269 | 5823 | 3220 | 1698 | 842 |

52,045 embryo transfers included. 7-year moving age groups are used. N is given for 7-year age group centered on age of interest. Age groups with fewer than 500 transfers are omitted.

## Supplemental Table 7: Best fit live birth rates based on day 5 fresh embryo morphology inner cell mass and trophectoderm quality.

|  | Best fit live birth rate per embryo | | | | | | | | | | | | |
| --- | --- | --- | --- | --- | --- | --- | --- | --- | --- | --- | --- | --- | --- |
| age | 30 | 31 | 32 | 32 | 33 | 34 | 34 | 35 | 36 | 37 | 37 | 38 | 39 |
| ICM Good, TE Good | 50% | 49% | 48% | 47% | 46% | 45% | 43% | 41% | 39% | 37% | 35% | 33% | 30% |
| ICM Good, TE Fair | 43% | 41% | 40% | 39% | 38% | 36% | 35% | 33% | 32% | 30% | 29% | 28% | 26% |
| ICM Good, TE Poor | 38% | 35% | 36% | 34% | 31% | 31% | 30% | 30% | 27% | 24% | 23% | 19% | 20% |
| ICM Fair, TE Good | 47% | 46% | 46% | 45% | 44% | 42% | 41% | 38% | 37% | 35% | 33% | 31% | 28% |
| ICM Fair, TE Fair | 37% | 37% | 35% | 34% | 33% | 32% | 30% | 29% | 27% | 25% | 23% | 21% | 19% |
| ICM Fair, TE Poor | 33% | 32% | 31% | 29% | 28% | 26% | 24% | 23% | 21% | 20% | 18% | 15% | 14% |
| ICM Poor, TE Good | 30% | 33% | 32% | 23% | 29% | 24% | 18% | 17% | 15% | 17% | 14% | 13% | 15% |
| ICM Poor, TE Fair | 31% | 28% | 22% | 18% | 18% | 16% | 15% | 16% | 15% | 11% | 10% | 12% | 12% |
| ICM Poor, TE Poor | 18% | 18% | 20% | 22% | 22% | 21% | 21% | 19% | 19% | 18% | 17% | 15% | 11% |
| n transfers | 26181 | 30535 | 34219 | 36662 | 37729 | 37302 | 35666 | 32943 | 29000 | 24570 | 19862 | 15366 | 11456 |
| n embryos | 33541 | 39229 | 44331 | 47967 | 50013 | 50320 | 48985 | 46178 | 41524 | 36054 | 30022 | 23894 | 18376 |

52,045 embryo transfers included. 9-year moving age groups are used. N is given for 9-year age group centered on age of interest. Data at the extremes of age has been omitted due to signs of overfittings in some groups.

## Supplemental Table 8: Best fit live birth rates based on day 5 frozen embryo morphology overall grade.

|  | Best fit live birth rate per embryo | | | | | | | | | | | | | | | | | | | | | | | | |
| --- | --- | --- | --- | --- | --- | --- | --- | --- | --- | --- | --- | --- | --- | --- | --- | --- | --- | --- | --- | --- | --- | --- | --- | --- | --- |
| Age | 23 | 24 | 25 | 26 | 27 | 27 | 28 | 29 | 30 | 30 | 31 | 32 | 33 | 34 | 34 | 35 | 36 | 37 | 38 | 39 | 39 | 40 | 41 | 42 | 43 |
| good | 50% | 49% | 50% | 49% | 49% | 49% | 48% | 48% | 47% | 47% | 46% | 45% | 44% | 43% | 42% | 40% | 38% | 36% | 33% | 30% | 27% | 23% | 20% | 19% | 16% |
| fair | 39% | 41% | 40% | 40% | 40% | 41% | 40% | 40% | 39% | 38% | 37% | 36% | 35% | 34% | 32% | 31% | 29% | 27% | 24% | 23% | 20% | 18% | 16% | 13% | 11% |
| Poor | 31% | 24% | 26% | 25% | 24% | 24% | 24% | 25% | 26% | 25% | 27% | 26% | 26% | 25% | 24% | 23% | 22% | 20% | 19% | 17% | 17% | 18% | 17% | 12% | 8% |
| n transfers | 1083 | 2111 | 3529 | 5794 | 9005 | 13010 | 17823 | 22777 | 27275 | 31361 | 34338 | 35594 | 34968 | 32701 | 29203 | 25179 | 20665 | 16002 | 11812 | 8459 | 5749 | 3781 | 2292 | 1302 | 714 |
| n embryos | 1484 | 2869 | 4744 | 7766 | 11940 | 17131 | 23288 | 29604 | 35313 | 40533 | 44354 | 46109 | 45579 | 42992 | 38875 | 34025 | 28440 | 22500 | 16994 | 12382 | 8585 | 5688 | 3462 | 1953 | 1039 |

56,891 embryo transfers included. 7-year moving age groups are used. N is given for 7-year age group centered on age of interest. Age groups with fewer than 500 transfers are omitted.

## Supplemental Table 9: Best fit live birth rates based on day 5 frozen embryo morphology expansion stage.

|  | Best fit live birth rate per embryo | | | | | | | | | | | | | | | | | | | | | | | | |
| --- | --- | --- | --- | --- | --- | --- | --- | --- | --- | --- | --- | --- | --- | --- | --- | --- | --- | --- | --- | --- | --- | --- | --- | --- | --- |
| age | 23 | 24 | 25 | 26 | 27 | 27 | 28 | 29 | 30 | 30 | 31 | 32 | 33 | 34 | 34 | 35 | 36 | 37 | 38 | 39 | 39 | 40 | 41 | 42 | 43 |
| early blast | 39% | 38% | 39% | 39% | 39% | 38% | 38% | 37% | 37% | 36% | 36% | 35% | 34% | 33% | 32% | 30% | 29% | 26% | 25% | 23% | 22% | 18% | 15% | 13% | 17% |
| expanded blast | 47% | 47% | 48% | 47% | 47% | 47% | 46% | 46% | 45% | 44% | 43% | 43% | 41% | 40% | 39% | 37% | 35% | 33% | 30% | 27% | 25% | 21% | 19% | 17% | 13% |
| hatching blast | 48% | 46% | 48% | 47% | 48% | 48% | 48% | 48% | 48% | 47% | 47% | 46% | 45% | 45% | 43% | 41% | 39% | 36% | 33% | 31% | 26% | 23% | 20% | 17% | 14% |
| n transfers | 1083 | 2111 | 3529 | 5794 | 9005 | 13010 | 17823 | 22777 | 27275 | 31361 | 34338 | 35594 | 34968 | 32701 | 29203 | 25179 | 20665 | 16002 | 11812 | 8459 | 5749 | 3781 | 2292 | 1302 | 714 |
| n embryos | 1484 | 2869 | 4744 | 7766 | 11940 | 17131 | 23288 | 29604 | 35313 | 40533 | 44354 | 46109 | 45579 | 42992 | 38875 | 34025 | 28440 | 22500 | 16994 | 12382 | 8585 | 5688 | 3462 | 1953 | 1039 |

56,891 embryo transfers included. 7-year moving age groups are used. N is given for 7-year age group centered on age of interest. Age groups with fewer than 500 transfers are omitted.

## Supplemental Table 10: Best fit live birth rates based on day 5 frozen embryo morphology inner cell mass quality.

|  | Best fit live birth rate per embryo | | | | | | | | | | | | | | | | | | | | | | | | |
| --- | --- | --- | --- | --- | --- | --- | --- | --- | --- | --- | --- | --- | --- | --- | --- | --- | --- | --- | --- | --- | --- | --- | --- | --- | --- |
| age | 23 | 24 | 25 | 26 | 27 | 27 | 28 | 29 | 30 | 30 | 31 | 32 | 33 | 34 | 34 | 35 | 36 | 37 | 38 | 39 | 39 | 40 | 41 | 42 | 43 |
| good | 50% | 50% | 51% | 50% | 49% | 50% | 49% | 49% | 48% | 47% | 47% | 46% | 45% | 43% | 42% | 40% | 38% | 36% | 32% | 30% | 27% | 23% | 20% | 18% | 15% |
| fair | 41% | 40% | 41% | 40% | 40% | 40% | 40% | 39% | 39% | 39% | 38% | 37% | 36% | 35% | 34% | 32% | 30% | 28% | 26% | 24% | 22% | 18% | 17% | 15% | 13% |
| poor | 22% | 28% | 30% | 29% | 27% | 27% | 27% | 27% | 25% | 24% | 26% | 26% | 25% | 25% | 24% | 24% | 25% | 21% | 21% | 19% | 17% | 18% | 19% | 12% | 9% |
| n transfers | 1083 | 2111 | 3529 | 5794 | 9005 | 13010 | 17823 | 22777 | 27275 | 31361 | 34338 | 35594 | 34968 | 32701 | 29203 | 25179 | 20665 | 16002 | 11812 | 8459 | 5749 | 3781 | 2292 | 1302 | 714 |
| n embryos | 1484 | 2869 | 4744 | 7766 | 11940 | 17131 | 23288 | 29604 | 35313 | 40533 | 44354 | 46109 | 45579 | 42992 | 38875 | 34025 | 28440 | 22500 | 16994 | 12382 | 8585 | 5688 | 3462 | 1953 | 1039 |

56,891 embryo transfers included. 7-year moving age groups are used. N is given for 7-year age group centered on age of interest. Age groups with fewer than 500 transfers are omitted.

## Supplemental Table 11: Best fit live birth rates based on day 5 frozen embryo morphology trophectoderm quality.

|  | Best fit live birth rate per embryo | | | | | | | | | | | | | | | | | | | | | | | | |
| --- | --- | --- | --- | --- | --- | --- | --- | --- | --- | --- | --- | --- | --- | --- | --- | --- | --- | --- | --- | --- | --- | --- | --- | --- | --- |
| age | 23 | 24 | 25 | 26 | 27 | 27 | 28 | 29 | 30 | 30 | 31 | 32 | 33 | 34 | 34 | 35 | 36 | 37 | 38 | 39 | 39 | 40 | 41 | 42 | 43 |
| good | 51% | 50% | 51% | 50% | 49% | 50% | 49% | 49% | 48% | 48% | 47% | 47% | 45% | 44% | 42% | 41% | 39% | 36% | 33% | 31% | 28% | 24% | 22% | 19% | 15% |
| fair | 41% | 42% | 42% | 42% | 42% | 42% | 42% | 41% | 40% | 40% | 39% | 38% | 37% | 35% | 34% | 33% | 30% | 28% | 26% | 24% | 22% | 18% | 15% | 14% | 14% |
| poor | 33% | 35% | 36% | 36% | 35% | 35% | 35% | 35% | 34% | 33% | 34% | 34% | 34% | 33% | 33% | 31% | 32% | 29% | 28% | 25% | 22% | 20% | 20% | 15% | 7% |
| n transfers | 1083 | 2111 | 3529 | 5794 | 9005 | 13010 | 17823 | 22777 | 27275 | 31361 | 34338 | 35589 | 34968 | 32701 | 29203 | 25179 | 20665 | 16002 | 11812 | 8459 | 5749 | 3781 | 2292 | 1302 | 714 |
| n embryos | 1484 | 2869 | 4744 | 7766 | 11940 | 17131 | 23288 | 29604 | 35313 | 40533 | 44354 | 46099 | 45579 | 42992 | 38875 | 34025 | 28440 | 22500 | 16994 | 12382 | 8585 | 5688 | 3462 | 1953 | 1039 |

56,891 embryo transfers included. 7-year moving age groups are used. N is given for 7-year age group centered on age of interest. Age groups with fewer than 500 transfers are omitted.

## Supplemental Table 12: Best fit live birth rates based on day 5 frozen embryo morphology inner cell mass and trophectoderm quality.

|  | Best fit live birth rate per embryo | | | | | | | | | | |
| --- | --- | --- | --- | --- | --- | --- | --- | --- | --- | --- | --- |
| age | 31 | 31 | 32 | 33 | 33 | 34 | 35 | 36 | 36 | 37 | 38 |
| ICM Good, TE Good | 48% | 47% | 46% | 45% | 44% | 43% | 41% | 40% | 38% | 36% | 33% |
| ICM Good, TE Fair | 44% | 43% | 42% | 41% | 40% | 38% | 37% | 35% | 34% | 31% | 29% |
| ICM Good, TE Poor | 46% | 43% | 43% | 43% | 42% | 40% | 39% | 37% | 37% | 38% | 39% |
| ICM Fair, TE Good | 43% | 43% | 43% | 42% | 42% | 41% | 40% | 39% | 36% | 35% | 33% |
| ICM Fair, TE Fair | 37% | 36% | 35% | 34% | 33% | 32% | 30% | 29% | 27% | 26% | 24% |
| ICM Fair, TE Poor | 36% | 37% | 37% | 37% | 36% | 35% | 34% | 33% | 33% | 31% | 28% |
| ICM Poor, TE Good | 47% | 47% | 45% | 38% | 34% | 32% | 34% | 32% | 27% | 19% | 14% |
| ICM Poor, TE Fair | 30% | 30% | 29% | 33% | 30% | 30% | 28% | 28% | 26% | 24% | 23% |
| ICM Poor, TE Poor | 25% | 23% | 23% | 23% | 22% | 22% | 22% | 23% | 23% | 20% | 20% |
| n transfers | 38054 | 41172 | 42418 | 41932 | 39785 | 36216 | 31707 | 26781 | 21623 | 16539 | 12087 |
| n embryos | 49296 | 53449 | 55262 | 54908 | 52467 | 48193 | 42645 | 36483 | 29910 | 23290 | 17384 |

56,891 embryo transfers included. 9-year moving age groups are used. N is given for 9-year age group centered on age of interest. Data at the extremes of age has been omitted due to signs of overfittings in some groups.

## Supplemental Table 13: Best fit live birth rates based on day 6 frozen embryo morphology overall grade.

|  | Best fit live birth rate per embryo | | | | | | | | | | | | | | | | | | | | | | |
| --- | --- | --- | --- | --- | --- | --- | --- | --- | --- | --- | --- | --- | --- | --- | --- | --- | --- | --- | --- | --- | --- | --- | --- |
| age | 24 | 25 | 26 | 27 | 28 | 28 | 29 | 30 | 31 | 31 | 32 | 33 | 34 | 35 | 35 | 36 | 37 | 38 | 39 | 40 | 40 | 41 | 42 |
| good | 39% | 40% | 41% | 40% | 40% | 41% | 40% | 40% | 40% | 39% | 39% | 38% | 36% | 35% | 33% | 32% | 29% | 27% | 24% | 21% | 18% | 16% | 12% |
| fair | 29% | 28% | 28% | 29% | 30% | 31% | 30% | 30% | 30% | 30% | 29% | 28% | 27% | 26% | 25% | 23% | 21% | 19% | 17% | 14% | 13% | 10% | 6% |
| Poor | 11% | 14% | 15% | 18% | 19% | 22% | 21% | 21% | 21% | 20% | 19% | 19% | 17% | 16% | 14% | 13% | 12% | 12% | 8% | 8% | 7% | 7% | 10% |
| n transfers | 549 | 967 | 1638 | 2712 | 4174 | 6006 | 7929 | 9830 | 11567 | 13111 | 13995 | 14195 | 13656 | 12655 | 11410 | 9863 | 8041 | 6288 | 4727 | 3413 | 2378 | 1474 | 871 |
| n embryos | 741 | 1312 | 2224 | 3646 | 5562 | 7934 | 10436 | 12905 | 15148 | 17086 | 18254 | 18614 | 18040 | 16867 | 15358 | 13402 | 11105 | 8810 | 6675 | 4836 | 3360 | 2070 | 1217 |

23,278 embryo transfers included. 7-year moving age groups are used. N is given for 7-year age group centered on age of interest. Age groups with fewer than 500 transfers are omitted.

## Supplemental Table 14: Best fit live birth rates based on day 6 frozen embryo morphology expansion stage.

|  | Best fit live birth rate per embryo | | | | | | | | | | | | | | | | | | | | | | |
| --- | --- | --- | --- | --- | --- | --- | --- | --- | --- | --- | --- | --- | --- | --- | --- | --- | --- | --- | --- | --- | --- | --- | --- |
| age | 24 | 25 | 26 | 27 | 28 | 28 | 29 | 30 | 31 | 31 | 32 | 33 | 34 | 35 | 35 | 36 | 37 | 38 | 39 | 40 | 40 | 41 | 42 |
| early blast | 27% | 28% | 30% | 25% | 25% | 26% | 25% | 24% | 23% | 22% | 23% | 21% | 20% | 19% | 19% | 18% | 17% | 15% | 15% | 13% | 12% | 9% | 7% |
| expanded blast | 34% | 32% | 33% | 35% | 34% | 36% | 35% | 35% | 35% | 34% | 34% | 33% | 32% | 30% | 29% | 27% | 24% | 21% | 19% | 16% | 13% | 11% | 9% |
| hatching blast | 38% | 39% | 39% | 39% | 41% | 41% | 40% | 40% | 40% | 39% | 38% | 37% | 36% | 35% | 33% | 31% | 29% | 28% | 25% | 23% | 20% | 17% | 10% |
| n transfers | 549 | 967 | 1638 | 2712 | 4174 | 6006 | 7929 | 9830 | 11567 | 13111 | 13995 | 14195 | 13656 | 12655 | 11410 | 9863 | 8041 | 6288 | 4727 | 3413 | 2378 | 1474 | 871 |
| n embryos | 741 | 1312 | 2224 | 3646 | 5562 | 7934 | 10436 | 12905 | 15148 | 17086 | 18254 | 18614 | 18040 | 16867 | 15358 | 13402 | 11105 | 8810 | 6675 | 4836 | 3360 | 2070 | 1217 |

23,278 embryo transfers included. 7-year moving age groups are used. N is given for 7-year age group centered on age of interest. Age groups with fewer than 500 transfers are omitted.

## Supplemental Table 15: Best fit live birth rates based on day 6 frozen embryo morphology inner cell mass quality.

|  | Best fit live birth rate per embryo | | | | | | | | | | | | | | | | | | | | | | |
| --- | --- | --- | --- | --- | --- | --- | --- | --- | --- | --- | --- | --- | --- | --- | --- | --- | --- | --- | --- | --- | --- | --- | --- |
| age | 24 | 25 | 26 | 27 | 28 | 28 | 29 | 30 | 31 | 31 | 32 | 33 | 34 | 35 | 35 | 36 | 37 | 38 | 39 | 40 | 40 | 41 | 42 |
| good | 39% | 39% | 40% | 40% | 40% | 40% | 39% | 39% | 39% | 39% | 39% | 38% | 37% | 35% | 34% | 32% | 30% | 27% | 25% | 22% | 19% | 17% | 14% |
| fair | 29% | 30% | 29% | 31% | 32% | 33% | 33% | 32% | 32% | 32% | 31% | 30% | 28% | 27% | 26% | 24% | 22% | 19% | 17% | 15% | 13% | 9% | 5% |
| Poor | 22% | 19% | 18% | 19% | 20% | 21% | 21% | 22% | 21% | 20% | 19% | 20% | 18% | 16% | 15% | 14% | 14% | 14% | 10% | 9% | 9% | 10% | 12% |
| n transfers | 549 | 967 | 1638 | 2712 | 4174 | 6006 | 7929 | 9830 | 11567 | 13111 | 13995 | 14195 | 13656 | 12655 | 11410 | 9863 | 8041 | 6288 | 4727 | 3413 | 2378 | 1474 | 871 |
| n embryos | 741 | 1312 | 2224 | 3646 | 5562 | 7934 | 10436 | 12905 | 15148 | 17086 | 18254 | 18614 | 18040 | 16867 | 15358 | 13402 | 11105 | 8810 | 6675 | 4836 | 3360 | 2070 | 1217 |

23,278 embryo transfers included. 7-year moving age groups are used. N is given for 7-year age group centered on age of interest. Age groups with fewer than 500 transfers are omitted.

## Supplemental Table 16: Best fit live birth rates based on day 6 frozen embryo morphology trophectoderm quality.

|  | Best fit live birth rate per embryo | | | | | | | | | | | | | | | | | | | | | | |
| --- | --- | --- | --- | --- | --- | --- | --- | --- | --- | --- | --- | --- | --- | --- | --- | --- | --- | --- | --- | --- | --- | --- | --- |
| age | 24 | 25 | 26 | 27 | 28 | 28 | 29 | 30 | 31 | 31 | 32 | 33 | 34 | 35 | 35 | 36 | 37 | 38 | 39 | 40 | 40 | 41 | 42 |
| good | 38% | 39% | 41% | 41% | 40% | 41% | 40% | 40% | 40% | 40% | 39% | 38% | 37% | 36% | 35% | 33% | 31% | 28% | 26% | 23% | 20% | 19% | 15% |
| fair | 34% | 33% | 30% | 32% | 33% | 34% | 34% | 33% | 33% | 32% | 32% | 31% | 29% | 27% | 26% | 24% | 22% | 20% | 17% | 15% | 14% | 10% | 7% |
| Poor | 15% | 18% | 23% | 24% | 23% | 23% | 24% | 25% | 26% | 24% | 23% | 24% | 24% | 22% | 21% | 19% | 17% | 16% | 14% | 11% | 8% | 6% | 6% |
| n transfers | 549 | 967 | 1638 | 2712 | 4174 | 6006 | 7929 | 9830 | 11567 | 13111 | 13995 | 14195 | 13656 | 12655 | 11410 | 9863 | 8041 | 6288 | 4727 | 3413 | 2378 | 1474 | 871 |
| n embryos | 741 | 1312 | 2224 | 3646 | 5562 | 7934 | 10436 | 12905 | 15148 | 17086 | 18254 | 18614 | 18040 | 16867 | 15358 | 13402 | 11105 | 8810 | 6675 | 4836 | 3360 | 2070 | 1217 |

23,278 embryo transfers included. 7-year moving age groups are used. N is given for 7-year age group centered on age of interest. Age groups with fewer than 500 transfers are omitted.

## Supplemental Table 17: Best fit live birth rates based on day 6 frozen embryo morphology inner cell mass and trophectoderm quality.

|  |  |  |  |  |  |  |  |  |  |  |  |  |  |
| --- | --- | --- | --- | --- | --- | --- | --- | --- | --- | --- | --- | --- | --- |
| age | 30 | 31 | 32 | 32 | 33 | 34 | 34 | 35 | 36 | 37 | 37 | 38 | 39 |
| ICM Good, TE Good | 40% | 40% | 40% | 39% | 38% | 38% | 36% | 35% | 34% | 33% | 31% | 29% | 27% |
| ICM Good, TE Fair | 37% | 36% | 35% | 35% | 34% | 32% | 31% | 29% | 28% | 26% | 24% | 22% | 20% |
| ICM Good, TE Poor | 26% | 26% | 32% | 30% | 29% | 29% | 29% | 26% | 23% | 24% | 23% | 11% | 11% |
| ICM Fair, TE Good | 39% | 38% | 38% | 37% | 36% | 34% | 33% | 32% | 29% | 28% | 27% | 24% | 22% |
| ICM Fair, TE Fair | 31% | 30% | 30% | 30% | 29% | 28% | 26% | 25% | 23% | 22% | 20% | 18% | 16% |
| ICM Fair, TE Poor | 29% | 28% | 27% | 25% | 26% | 25% | 25% | 24% | 22% | 19% | 18% | 18% | 17% |
| ICM Poor, TE Good | 23% | 19% | 23% | 25% | 30% | 28% | 27% | 25% | 25% | 23% | 30% | 26% | 22% |
| ICM Poor, TE Fair | 21% | 20% | 21% | 22% | 20% | 19% | 19% | 17% | 16% | 17% | 17% | 16% | 13% |
| ICM Poor, TE Poor | 20% | 19% | 18% | 20% | 19% | 18% | 15% | 14% | 13% | 13% | 13% | 13% | 7% |
| n transfers | 12251 | 14214 | 15768 | 16793 | 17065 | 16622 | 15635 | 14185 | 12425 | 10489 | 8409 | 6483 | 4805 |
| n embryos | 16073 | 18587 | 20624 | 22037 | 22492 | 22033 | 20862 | 19039 | 16787 | 14286 | 11626 | 9074 | 6779 |

23,278 embryo transfers included. 9-year moving age groups are used. N is given for 9-year age group centered on age of interest. Data at the extremes of age has been omitted due to signs of overfittings in some groups.

## Supplemental Table 18: Best fit live birth rates based on day 3 cleavage stage embryo morphology for fresh embryo transfers.

|  | Best fit live birth rate per embryo | | | | | | | | | | | | | | | | | | | | | |
| --- | --- | --- | --- | --- | --- | --- | --- | --- | --- | --- | --- | --- | --- | --- | --- | --- | --- | --- | --- | --- | --- | --- |
| age | 27 | 28 | 28 | 29 | 30 | 31 | 32 | 33 | 34 | 34 | 35 | 36 | 37 | 38 | 39 | 40 | 40 | 41 | 42 | 43 | 43 | 44 |
| 4 cell | 5% | 9% | 11% | 9% | 6% | 5% | 4% | 3% | 2% | 1% | 1% | 1% | 1% | 1% | 0% | 1% | 1% | 0% | 1% | 1% | 0% | 0% |
| 5 cell | 7% | 11% | 7% | 8% | 10% | 7% | 7% | 6% | 6% | 5% | 5% | 3% | 3% | 2% | 2% | 1% | 1% | 1% | 1% | 1% | 1% | 1% |
| 6 cell | 11% | 9% | 9% | 10% | 10% | 11% | 10% | 9% | 8% | 8% | 7% | 7% | 6% | 5% | 5% | 4% | 4% | 3% | 1% | 0% | 0% | -1% |
| 7 cell | 25% | 21% | 20% | 19% | 18% | 19% | 18% | 17% | 17% | 17% | 16% | 14% | 12% | 11% | 10% | 9% | 7% | 6% | 5% | 4% | 3% | 3% |
| 8 cell | 30% | 30% | 30% | 29% | 29% | 29% | 28% | 27% | 26% | 24% | 23% | 21% | 19% | 17% | 15% | 13% | 11% | 9% | 7% | 5% | 4% | 2% |
| >8 cell | 30% | 27% | 29% | 26% | 26% | 26% | 25% | 25% | 24% | 23% | 22% | 20% | 18% | 17% | 15% | 13% | 11% | 9% | 7% | 5% | 3% | 2% |
| n transfers | 1207 | 1853 | 2841 | 3922 | 5245 | 6748 | 8462 | 10204 | 11942 | 13251 | 14522 | 15531 | 16026 | 15867 | 15034 | 13617 | 12015 | 9973 | 7693 | 5583 | 3800 | 2414 |
| n embryos | 2054 | 3151 | 4849 | 6696 | 9007 | 11600 | 14593 | 17732 | 21027 | 23655 | 26546 | 29092 | 30723 | 30971 | 29785 | 27299 | 24235 | 20079 | 15305 | 10873 | 7238 | 4479 |

28,878 embryo transfers included. 7-year moving age groups are used. N is given for 7-year age group centered on age of interest. Age groups with fewer than 1,000 transfers are omitted.

## Supplemental Table 19: Best fit live birth rates based on day 3 cleavage stage embryo morphology for fresh transfers with analysis of 8-cell embryo fragmentation.

|  | Best fit live birth rate per embryo | | | | | | | | | | | | | | | | | | | |
| --- | --- | --- | --- | --- | --- | --- | --- | --- | --- | --- | --- | --- | --- | --- | --- | --- | --- | --- | --- | --- |
| age | 28 | 29 | 30 | 31 | 32 | 33 | 34 | 34 | 35 | 36 | 37 | 38 | 39 | 39 | 40 | 41 | 42 | 43 | 43 | 44 |
| 4 cell | 11% | 8% | 6% | 5% | 4% | 4% | 2% | 1% | 2% | 1% | 1% | 1% | 1% | 1% | 1% | 1% | 1% | 1% | 0% | 0% |
| 5 cell | 7% | 8% | 10% | 8% | 7% | 6% | 6% | 6% | 5% | 3% | 3% | 2% | 2% | 2% | 1% | 1% | 1% | 1% | 1% | 1% |
| 6 cell | 10% | 11% | 11% | 11% | 11% | 9% | 9% | 8% | 8% | 7% | 6% | 5% | 5% | 5% | 4% | 3% | 1% | 0% | 0% | -1% |
| 7 cell | 20% | 19% | 17% | 19% | 18% | 18% | 18% | 17% | 16% | 14% | 12% | 11% | 10% | 9% | 7% | 6% | 5% | 4% | 3% | 3% |
| 8 cell 0% | 31% | 29% | 29% | 30% | 29% | 28% | 27% | 25% | 24% | 22% | 19% | 17% | 15% | 13% | 11% | 9% | 7% | 4% | 3% | 1% |
| 8 cell 1-10% | 31% | 30% | 31% | 29% | 28% | 27% | 26% | 25% | 23% | 21% | 19% | 17% | 15% | 14% | 12% | 10% | 9% | 7% | 6% | 3% |
| 8 cell >10% | 22% | 23% | 22% | 21% | 20% | 20% | 19% | 18% | 16% | 15% | 13% | 12% | 10% | 8% | 7% | 6% | 4% | 2% | 1% | 0% |
| >8 cell | 28% | 26% | 26% | 26% | 25% | 25% | 25% | 23% | 22% | 20% | 18% | 17% | 14% | 12% | 11% | 9% | 7% | 5% | 3% | 3% |
| n transfers | 2635 | 3650 | 4858 | 6240 | 7816 | 9415 | 11011 | 12232 | 13416 | 14363 | 14825 | 14682 | 13901 | 12593 | 11109 | 9174 | 7071 | 5120 | 3472 | 2209 |
| n embryos | 4463 | 6191 | 8284 | 10645 | 13367 | 16218 | 19211 | 21635 | 24304 | 26639 | 28139 | 28374 | 27270 | 24991 | 22172 | 18264 | 13914 | 9853 | 6525 | 4032 |

26,672 embryo transfers included. 7-year moving age groups are used. N is given for 7-year age group centered on age of interest. Age groups with fewer than 2,000 transfers are omitted.

## Supplemental Table 20: Best fit live birth rates based on day 3 cleavage stage embryo morphology for frozen embryo transfers.

|  | Best fit live birth rate per embryo | | | | | | | | | | | | | | |
| --- | --- | --- | --- | --- | --- | --- | --- | --- | --- | --- | --- | --- | --- | --- | --- |
| age | 29 | 30 | 31 | 31 | 32 | 33 | 34 | 35 | 36 | 37 | 38 | 38 | 39 | 40 | 41 |
| 4 cell | 19% | 16% | 15% | 13% | 10% | 7% | 7% | 8% | 9% | 7% | 8% | 8% | 8% | 7% | 2% |
| 5 cell | 12% | 13% | 12% | 11% | 12% | 10% | 8% | 5% | 4% | 3% | 4% | 2% | 1% | 2% | 3% |
| 6 cell | 14% | 14% | 14% | 15% | 12% | 10% | 10% | 9% | 7% | 6% | 4% | 4% | 3% | 2% | 2% |
| 7 cell | 27% | 24% | 23% | 21% | 21% | 18% | 17% | 12% | 11% | 8% | 7% | 5% | 5% | 3% | 4% |
| 8 cell | 26% | 25% | 25% | 25% | 24% | 23% | 21% | 20% | 19% | 17% | 14% | 12% | 10% | 7% | 6% |
| >8 cell | 21% | 22% | 21% | 21% | 20% | 19% | 19% | 17% | 15% | 14% | 12% | 11% | 10% | 8% | 6% |
| n transfers | 1179 | 1457 | 1777 | 2060 | 2295 | 2476 | 2564 | 2667 | 2666 | 2558 | 2389 | 2131 | 1859 | 1590 | 1274 |
| n embryos | 2058 | 2531 | 3094 | 3585 | 3988 | 4325 | 4510 | 4752 | 4819 | 4711 | 4467 | 4070 | 3607 | 3104 | 2479 |

5,250 embryo transfers included. 7-year moving age groups are used. N is given for 7-year age group centered on age of interest. Age groups with fewer than 1,000 transfers are omitted.
