# Supplementary material for: Embryo morphology and live birth in the United States
Source: F S Rep. 2022 Feb 23;3(2):131–7. doi: 10.1016/j.xfre.2022.02.006 (PMC9250116; doi:10.1016/j.xfre.2022.02.006)
Supplement: Legend for Figure [file mmc4.docx]

Supplemental Figures

## Supplemental Figure 1. Logic for predicting outcomes of a double embryo transfer incorporating a universal factors fraction and live birth rates for embryo 1 and embryo 2.

The universal factors fraction (UNI) is a fraction from 0 (never favorable) to 1 (always favorable). The probability of each outcome is equal to the product of the terms next to the corresponding arrows above and the corresponding terms on the perimeter of the square. For example, the probability of twins is UNI x $\frac{\mathrm{LBR}e1}{\mathrm{UNI}}$ x $\frac{\mathrm{LBR}e2}{\mathrm{UNI}}$. This same logic can be applied to transfer of more than two embryos. A UNI value of 0.70, representing adequate universal factors (such as uterine receptivity) 70% of the time, can be used as a best estimate for both fresh and frozen embryo transfers. UNI, universal factors fraction; LBRe1, live birth rate for embryo 1; LBRe2, live birth rate for embryo 2. This figure has been previously published under a Creative Commons license (Awadalla M, Vestal N, McGinnis L, Ahmady A. Effect of Age and Morphology on Live Birth Rate After Cleavage Stage Embryo Transfer. Reprod Sci 2021;43–51. https://doi.org/10.1007/s43032-020-00249-9).

## Supplemental Figure 2. Best fit live birth rates per embryo for day 3 embryo transfers comparing single and multiple embryo transfers.

5-year moving age groups are used for this analysis.

## Supplemental Figure 3. Best fit live birth rates per embryo for day 5 fresh embryo transfers comparing single and multiple embryo transfers.

5-year moving age groups are used for this analysis.

##

## Supplemental Figure 4. Best fit live birth rates per embryo for day 5 frozen embryo transfers comparing single and multiple embryo transfers.

5-year moving age groups are used for this analysis.

## Supplemental Figure 5. Embryo morphology distribution for day 3 embryos.

## Supplemental Figure 6. Embryo morphology distribution for day 5 embryos.

**Supplemental Figure 7. Best fit live birth rates based on day 5 fresh embryo morphology expansion stage.**

##

## Supplemental Figure 8. Best fit live birth rates based on day 5 fresh embryo morphology inner cell mass quality.

## Supplemental Figure 9. Best fit live birth rates based on day 5 fresh embryo morphology trophectoderm quality.

## Supplemental Figure 10. Best fit live birth rates based on day 5 fresh embryo morphology inner cell mass and trophectoderm quality.

**Supplemental Figure 11. Best fit live birth rates based on day 5 frozen embryo morphology expansion stage.**

## Supplemental Figure 12. Best fit live birth rates based on day 5 frozen embryo morphology inner cell mass quality.

## Supplemental Figure 13. Best fit live birth rates based on day 5 frozen embryo morphology trophectoderm quality.

**Supplemental Figure 14. Best fit live birth rates based on day 5 frozen embryo morphology inner cell mass and trophectoderm quality.**

**Supplemental Figure 15. Best fit live birth rates based on day 6 frozen embryo morphology expansion stage.**

## Supplemental Figure 16. Best fit live birth rates based on day 6 frozen embryo morphology inner cell mass quality.

## Supplemental Figure 17. Best fit live birth rates based on day 6 frozen embryo morphology trophectoderm quality.

**Supplemental Figure 18. Best fit live birth rates based on day 6 frozen embryo morphology inner cell mass and trophectoderm quality.**

**Supplemental Figure 19. Predicted transfer outcomes by average live birth rate per embryo and number of embryos for a universal factors fraction of 0.70.** The multiples column results are shaded green, yellow, red, or gray to indicate the risk of multiples at delivery (0–9%, 10–19%, 20–29%, or ≥ 30% respectively). LBR, total live birth rate per embryo transfer; % mult., percentage of live deliveries that are multiples; % twins, percentage of live deliveries that are twin deliveries; % trip. or >, percentage of live deliveries that are triplets or greater. This figure has been previously published under a Creative Commons license (Awadalla M, Vestal N, McGinnis L, Ahmady A. Effect of Age and Morphology on Live Birth Rate After Cleavage Stage Embryo Transfer. Reprod Sci 2021;43–51. https://doi.org/10.1007/s43032-020-00249-9).

**Supplemental Figure 20. Predicted and actual rates of multiples for day 3 fresh embryo transfer.**

Predicted rates of multiples are based on maternal age, best fit live birth rate per embryo, universal factors fraction of 0.70, and without considering embryo morphology. Rates of multiples are given as number of deliveries of multiples divided by total number of deliveries**.**

## Supplemental Figure 21. Predicted and actual rates of multiples for day 5 fresh embryo transfer.

Predicted rates of multiples are based on maternal age, best fit live birth rate per embryo, universal factors fraction of 0.70, and without considering embryo morphology. Rates of multiples are given as number of deliveries of multiples divided by total number of deliveries.

## Supplemental Figure 22. Predicted and actual rates of multiples for day 5 frozen embryo transfer.

Predicted rates of multiples are based on maternal age, best fit live birth rate per embryo, universal factors fraction of 0.70, and without considering embryo morphology. Rates of multiples are given as number of deliveries of multiples divided by total number of deliveries.

**Supplemental Figure 23. Live birth rate per embryo is not linear on the logit scale.**
